# Amino acid metabolism in breast cancer: pathogenic drivers and therapeutic opportunities

**DOI:** 10.1093/procel/pwaf011

**Published:** 2025-02-20

**Authors:** Yawen Liu, Xiangyun Zong, Patricia Altea-Manzano, Jie Fu

**Affiliations:** Department of Radiation Oncology, Shanghai Sixth People’s Hospital, Affiliated to Shanghai Jiao Tong University School of Medicine, Shanghai 200233, China; Department of Breast Surgery, Shanghai Sixth People’s Hospital, Affiliated to Shanghai Jiao Tong University School of Medicine, Shanghai 200233, China; Andalusian Molecular Biology and Regenerative Medicine Centre (CABIMER), Universidad de Sevilla-CSIC-Universidad Pablo de Olavide, Seville 41004, Spain; Department of Radiation Oncology, Shanghai Sixth People’s Hospital, Affiliated to Shanghai Jiao Tong University School of Medicine, Shanghai 200233, China

**Keywords:** amino acid metabolism, breast cancer, metabolic reprogramming, cancer therapy, personalized medicine

## Abstract

Amino acid metabolism plays a critical role in the progression and development of breast cancer. Cancer cells, including those in breast cancer, reprogram amino acid metabolism to meet the demands of rapid proliferation, survival, and immune evasion. This includes alterations in the uptake and utilization of amino acids, such as glutamine, serine, glycine, and arginine, which provide essential building blocks for biosynthesis, energy production, and redox homeostasis. Notably, the metabolic phenotypes of breast cancer cells vary across molecular subtypes and disease stages, emphasizing the need for patient stratification and personalized therapeutic strategies. Advances in multi-level diagnostics, including phenotyping and predictive tools, such as AI-based analysis and body fluid profiling, have highlighted the potential for tailoring treatments to individual metabolic profiles. Enzymes, such as glutaminase and serine hydroxymethyltransferase, often upregulated in breast cancer, represent promising therapeutic targets. Understanding the interplay between amino acid metabolism and breast cancer biology, alongside the integration of personalized medicine approaches, can uncover novel insights into tumor progression and guide the development of precision therapies. This review explores the metabolic pathways of amino acids in breast cancer, with a focus on their implications for personalized treatment strategies.

## Introduction

Breast cancer, a heterogeneous and multifaceted disease, represents one of the most prevalent malignancies affecting women worldwide (Bray et al., 2024). Based on gene expression profiles and the presence of specific biomarkers, breast cancer can be classified into the following major molecular subtypes: Luminal A, Luminal B (HER2-negative), Luminal B (HER2-positive), HER2-positive, and basal-like tumor breast cancers (mainly triple-negative, TNBC). In addition to surgical treatment, endocrine therapy (hormone therapy) is the main treatment for Luminal A, Luminal B (HER2-negative) type. Luminal B (HER2-positive) and HER2-positive types are highly aggressive due to amplification or overexpression of the *HER2* gene, but targeted therapies against HER2 have dramatically improved prognosis in this subtype. Triple-negative breast cancer (negative for ER, PR and HER2) accounts for about 10%–15% of breast cancers and is characterized by strong aggressiveness and high recurrence rate, and its lack of clear targeted therapeutic targets has led to poor prognosis and shorter overall survival in TNBC patients (Loibl et al., 2024). Molecular typing of breast cancer has become an important basis for the development of individualized treatment plans, and with the progress of targeted therapy and immunotherapy, the survival rate and quality of life of patients have been significantly improved. However, ongoing research is still needed to address challenges such as treatment resistance and recurrence to further optimize breast cancer treatment strategies. To address these limitations, critical biological processes such as nutrient metabolism are being investigated in breast cancer with the aim of discovering novel targeted therapies.

One of the hallmarks of cancer metabolism is the reprogramming of nutrient uptake and utilization to meet the energy and biosynthetic needs of cancer cells (You et al., 2023). To meet the requirements of rapid proliferation and replication, breast cancer cells require a large supply of macronutrients and therefore their energy and biosynthetic requirements are extremely high. In fact, to provide precursors to biosynthetic pathways, cancer cells reprogram their metabolism as well as upregulate the uptake and catabolism of certain nutrients (Jiao et al., 2023). Metabolism of amino acids, nutrients vital to the survival of all cell types, is one of the mechanisms that cancer cells exploit to match their needs. Amino acids are essential not only for biosynthesis, energy production, and maintaining redox balance in malignant cancer cells but also as key metabolites that support immune cell activation and promote antitumor activity within the tumor microenvironment (Lieu et al., 2020).

This metabolic reprogramming has been widely reported in breast cancer, where alterations in amino acid transport and metabolism are closely linked to tumor growth and survival. Breast cancer cells exhibit increased uptake and utilization of specific amino acids (e.g., glutamine, serine, and arginine), which are not only substrates for protein synthesis, but also play key roles in cellular signaling, energy production, and maintenance of redox balance (Parida et al., 2022; Tombari et al., 2023). These metabolic adaptations are often driven by oncogenes and tumor suppressors, including c-Myc, mTOR, and p53, which orchestrate the expression and activity of key enzymes and transport proteins involved in amino acid metabolism (Colombero et al., 2021; Shajahan-Haq et al., 2014).

Key transporters, such as SLC1A5 (also known as ASCT2) and SLC7A5 (LAT1), facilitate glutamine and essential amino acid uptake, respectively, and are upregulated in various breast cancer subtypes. These transporters, as well as enzymes such as glutaminase (GLS) and phosphoglycerate dehydrogenase (PHGDH), contribute to the metabolic flexibility and adaptability of cancer cells, allowing them to thrive in nutrient-deficient tumor microenvironments (Sullivan et al., 2019; Vidula et al., 2023). In addition, interactions between amino acid metabolism and signaling pathways, such as the mTOR pathway, further emphasize the importance of reprogramming amino acid metabolism in regulating breast cancer cell growth, proliferation, and survival. In addition to supporting anabolic processes, amino acid metabolism in breast cancer is closely linked to the regulation of oxidative stress and apoptosis. For example, glutamine is a precursor for the synthesis of glutathione, a major cellular antioxidant that helps to mitigate oxidative damage and promote cell survival. The dependence of breast cancer cells on specific amino acids and their metabolic pathways highlights potential therapeutic opportunities. Targeting amino acid transporters, enzymes, and related signaling pathways is expected to lead to the development of novel anti-cancer strategies.

The aim of this review is to provide an overview of the importance of amino acid metabolism in breast cancer, to explore how metabolic reprogramming supports tumor growth and survival, and to discuss the potential for therapeutic interventions targeting these metabolic pathways. By delving into the intricate relationship between amino acid metabolism and breast cancer biology, we can better understand the complexity of the disease and find new avenues for treatment.

## Amino acid metabolism in breast cancer

### Glutamine metabolism

**Figure 1.**
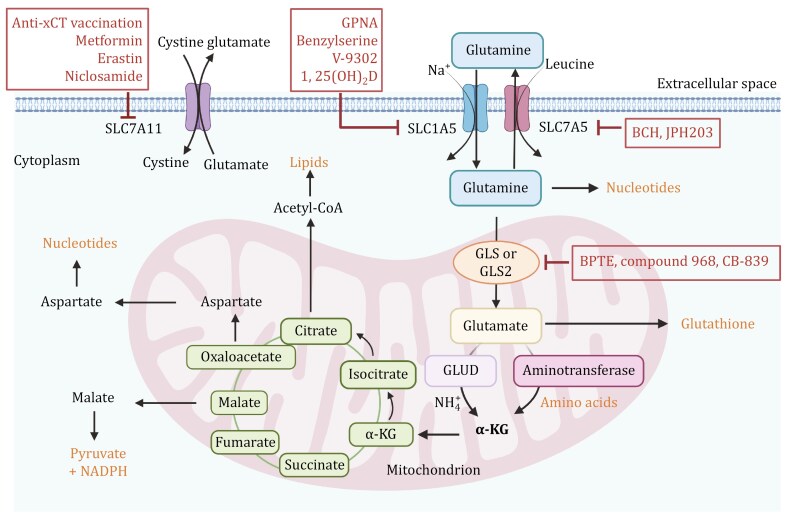
**Glutamine metabolic pathways.** Glutamine is transported into the cell via transporters SLC1A5 and SLC7A5. Once inside the cell, glutamine can be converted into glutamate by glutaminase (GLS or GLS2). Glutamate then contributes to the production of α-ketoglutarate (α-KG) via glutamate dehydrogenase (GLUD) or aminotransferases, feeding into the tricarboxylic acid (TCA) cycle. This pathway is critical for producing energy, biosynthetic precursors, and maintaining redox balance. Therapeutic Targets: Inhibition of glutaminase by compounds such as BPTES, compound 968, and CB-839 can reduce glutamine metabolism, potentially impairing cancer cell growth. SLC1A5 and SLC7A5 inhibitors include GPNA, Benzylserine, V-9302, and 1,25(OH)_2_D. Inhibitors such as BCH and JPH203 target SLC7A5. Targeting SLC7A11 with inhibitors like Anti-xCT, Metformin, Erastin and Niclosamide disrupts cystine and glutamate balance, impacting cancer cell survival. The figure was created with BioRender.com.

Glutamine is the most abundant circulating amino acid and plays a critical and diverse role in cancer cells, providing not only a nitrogen source for amino acid and nucleotide biosynthesis but also a carbon source to replenish the TCA cycle and lipid biosynthetic pathways (Altman et al., 2016; Yoo et al., 2020) (Fig. 1). Rapidly proliferating cancer cells then exhibit increased glutamine uptake and glutamine dependence, termed “glutamine addiction” (Quek et al., 2022). In fact, cancer cells maintain high intracellular biosynthesis levels by accelerating the intracellular glutamine metabolism to replenish metabolic intermediates into the TCA cycle, a process known as glutamine anaplerosis. Glutamine can be converted to glutamate by glutaminase (GLS), which is then catalyzed by glutamate dehydrogenase (GLUD) or aminotransferase to generate α-ketoglutarate (α-KG) into the TCA cycle, thus providing cells with energy and intermediate metabolites (Wang et al., 2020b). GLS is a key enzyme in glutamine catabolism and serves as the rate-limiting step in the glutamine anaplerosis pathway (Jin et al., 2023). There are two main isoforms of this enzyme: kidney-type GLS (GLS1) and liver-type GLS (GLS2), both of which are localized in mitochondria (Feng et al., 2024). These isoforms have markedly different expression patterns. GLS1 is typically upregulated in cancer and is associated with tumor growth and proliferation, playing a critical role in many types of cancer. On the other hand, GLS2 expression is usually suppressed and can act as a tumor suppressor depending on the environmental context (Sun et al., 2020). However, it has been demonstrated that knocking down GLS2 in breast cancer cells can reduce cell growth and glutamine metabolism-related phenotypes (Dias et al., 2020). When breast cancer cells with high GLS2 expression and low GLS1 expression are injected into the flanks of NOD/SCID mice, there is a significant increase in the number of metastatic lesions in the lungs, indicating that GLS2 may promote breast cancer metastasis, potentially dependent on low GLS1 levels, although the specific mechanism remains unclear (Dias et al., 2020). The expression of GLS varies among different molecular subtypes of breast cancer. GLS2 levels are relatively high in Luminal A and Luminal B breast cancers, while GLS1 levels are higher in TNBC, with a significant negative correlation between the expressions of GLS1 and GLS2. Both glutaminase isoforms have low expression in HER2-positive breast tumors (Vidula et al., 2023). Compared to ER-positive breast cancer, HER2-positive breast cancer and TNBC show higher levels of glutamate and lower levels of glutamine (Masisi et al., 2021), suggesting that these subtypes may have higher glutamine influx and active glutaminolysis. The expression of GLS isoforms in breast cancer can be regulated by different genes. c-Myc is a key driver of maintaining the glutaminolysis phenotype and can enhance the expression of mitochondrial GLS leading to uncontrolled proliferation of tumor cells (Gao et al., 2009). Additionally, ErbB2 activation can upregulate GLS1 expression in breast cancer cells through a mechanism controlled by the NF-κB pathway rather than by c-Myc, promoting glutamine utilization in cancer cells (Qie et al., 2014). The expression of the *GLS2* gene is partially driven by the transcription factor GATA3, a major regulator of luminal differentiation, suggesting that high levels of GLS2 may be intrinsically linked to the luminal cell state (Lukey et al., 2019).

Glutamine-derived glutamate is primarily converted into α-KG through two main pathways. The first pathway involves the reversible deamination of glutamate catalyzed by GLUD1/2, which produces α-KG and releases ammonium. The GLUD pathway generates NADH and NADPH, which participate in the TCA cycle, control of ROS levels, and lipid synthesis (Yoo et al., 2020). The second pathway involves transaminases (such as glutamate-oxaloacetate transaminase (GOT), glutamate-pyruvate transaminase (GPT), and phosphoserine aminotransferase (PSAT)) that convert glutamate into α-KG without producing ammonia. GOT transfers nitrogen from glutamate to oxaloacetate, producing aspartate and α-KG. GPT transfers nitrogen from glutamate to pyruvate, producing alanine and α-KG. PSAT, which is part of the serine biosynthesis pathway, transfers nitrogen from glutamate to 3-phosphohydroxy pyruvate, producing phosphoserine and α-KG. The transaminase pathway also generates other amino acids such as aspartate, alanine, and serine, which contribute to various cellular functions (Jin et al., 2023). Thus, these two pathways are in competition within the cell. Interestingly, research has shown that mammary epithelial cells shift between proliferative and quiescent states during development, the menstrual cycle, pregnancy, and carcinogenesis (Coloff et al., 2016). Mammary epithelial cells in different states use different glutamate metabolism pathways: highly proliferative breast tumors exhibit high transaminase and low GLUD expression, preferring to convert glutamate to α-KG via transaminases to synthesize non-essential amino acids, whereas quiescent cells rely more on GLUD to produce α-KG (Coloff et al., 2016).

Among the four main breast cancer subtypes, the highly proliferative basal-like tumors typically exhibit high levels of GPT2 and PSAT1 expression, while GLUD1/2 expression is relatively low (Sun et al., 2020). In contrast, ER-positive breast cancers show increased GLUD expression, explaining their relative independence from glutamine (Sun et al., 2020). Additionally, luminal breast cancers have lower glutamine dependency, partly due to the luminal transcription factor GATA3, which can directly induce high expression of glutamine synthetase (GS), catalyzing the ATP-dependent reaction for *de novo* glutamine synthesis (Issaq et al., 2019; Kim et al., 2021). Furthermore, GOT2 is also overexpressed in TNBC, promoting cell proliferation by increasing the production of aspartate and α-KG. The BRCA1 protein suppresses GOT2 expression at the transcriptional level, but this inhibitory mechanism is weakened due to the frequent BRCA1 deficiency observed in TNBC (Hong et al., 2019).

Finally, α-KG derived from glutamine in tumor cells typically participates in the TCA cycle in two ways, depending mainly on oxygen supply and mitochondrial function. Under aerobic conditions, tumor cells with normal mitochondrial function can use α-KG in the TCA cycle, thereby providing energy and biosynthetic precursors for cell growth and proliferation. Conversely, under hypoxic conditions or when mitochondrial function is impared, α-KG primarily participates in lipid synthesis through reductive carboxylation (Cluntun et al., 2017).

### Serine metabolism

**Figure 2.**
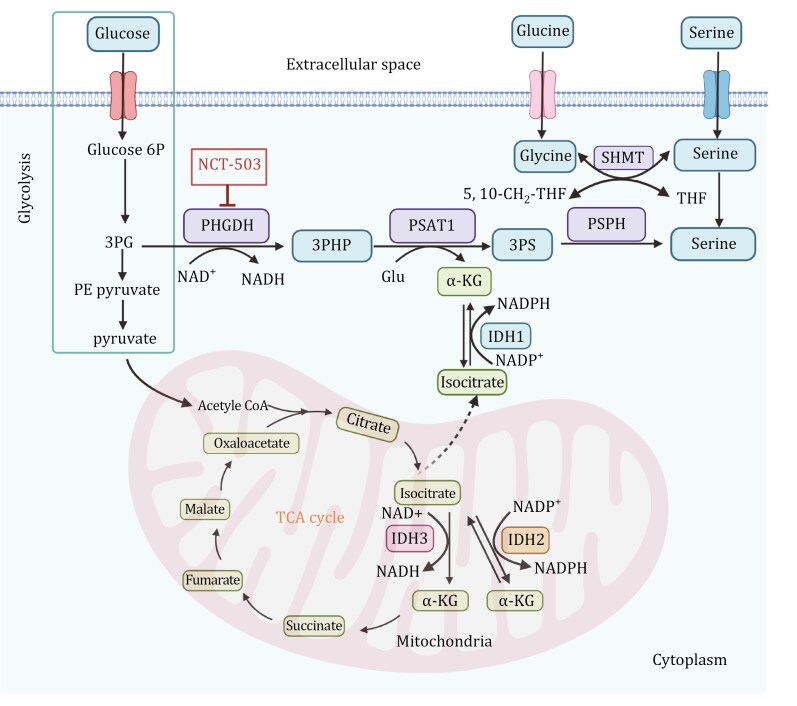
**Serine and glycine biosynthesis pathways and the link with mitochondria metabolism.** Glucose enters the cell and is metabolized through glycolysis, producing Glucose 6P, 3PG, and pyruvate. 3PG is diverted into the serine biosynthesis pathway, catalyzed by PHGDH, PSAT1, and PSPH, resulting in the production of serine. Serine can be directly utilized, while glycine can be synthesized from serine through the action of SHMT, which facilitates the reversible conversion between serine and glycine. α-KG, derived from glutamate, fuels the TCA cycle. The mitochondrial TCA cycle integrates oxidative and reductive metabolic pathways, involving IDH1, IDH2, and IDH3. IDH3 is NAD^+^-dependent, catalyzing the oxidative decarboxylation of isocitrate to α-KG, generating NADH and producing energy. IDH2, utilizing NADP^+^, facilitates the bidirectional metabolism of isocitrate and α-KG, promoting the reductive carboxylation of α-KG to citrate under specific conditions such as hypoxia or mitochondrial dysfunction. In the cytoplasm, IDH1, which also relies on NADP^+^, catalyzes the same reductive carboxylation reaction, supporting redox balance and lipid biosynthesis. The therapeutic inhibitor NCT-503 targets PHGDH, disrupting serine biosynthesis and impeding tumor growth by exploiting the dependence of cancer cells on this pathway. This highlights the metabolic crosstalk between serine/glycine biosynthesis, glycolysis, and the TCA cycle, emphasizing the involvement of mitochondria in the metabolic reprogramming of cancer cells. Glucose 6P: glucose-6-phosphate; 3PG: 3-phosphoglycerate; 3PHP: 3-phosphohydroxypyruvate; PHGDH: phosphoglycerate dehydrogenase; 3PS: 3-phosphoserine; PSAT1: phosphoserine aminotransferase 1; Glu: glutamate; α-KG: α-ketoglutarate; PSPH: phosphoserine phosphatase; SHMT: serine hydroxymethyltransferase; THF: tetrahydrofolate; IDH: isocitrate dehydrogenase. The figure was created with BioRender.com.

Among amino acids, serine is the second most consumed after glutamine. Serine is an important non-essential amino acid that can be obtained from the diet or synthesized *de novo* from the glycolysis intermediate 3-phosphoglycerate (3-PG) (Wu et al., 2017) (Fig. 2). It serves multiple purposes in tumor cells: it is used for protein synthesis, acts as a precursor for glycine and cysteine in the synthesis of sphingolipids and phospholipids, and is involved in the folate-mediated one-carbon pathway, where it is cleaved to produce glycine and one-carbon units for the synthesis of porphyrins, thymidylate, purines, glutathione, and S-adenosylmethionine (SAM) (Barnabas et al., 2021). Serine metabolism not only provides essential precursors for the synthesis of proteins, nucleic acids, and lipids within tumor cells but also contributes to maintaining redox balance by supplying reducing power.

PHGDH is the first enzyme in the serine biosynthesis pathway. It diverts the glycolytic pathway by catalyzing the conversion of 3-phosphoglycerate (3-PG) to 3-phosphohydroxypyruvate (3-PHP). 3-PHP is then transaminated to 3-phosphoserine by phosphoserine aminotransferase 1 (PSAT1) and subsequently dephosphorylated to serine by phosphoserine phosphatase (PSPH) (Barnabas et al., 2021) (Fig. 2). Serine and glycine are involved in one-carbon metabolism, supporting the production of NADPH and the biosynthesis of nucleotides and glutathione. Additionally, serine metabolism is intricately linked to the TCA cycle, as the TCA intermediate α-KG is produced during the PSAT1 transamination reaction and can enter the cycle or support other metabolic pathways. Furthermore, reductive carboxylation, driven by isocitrate dehydrogenase, can regenerate α-KG from citrate under hypoxic or nutrient-limiting conditions, sustaining serine biosynthesis and maintaining redox balance through NADPH production. This interplay highlights the metabolic flexibility required for cellular proliferation and survival under stress conditions.

Studies have found an increase in serine synthesis within breast cancer tissues, along with an upregulation of the serine transporter protein SLC1A4 (Pollari et al., 2011; Possemato et al., 2011). In breast cancer cells with LKB1 deficiency, there is an upregulation of PSAT1, PSPH, and serine hydroxymethyltransferase (SHMT1/2), all of which are involved in the *de novo* serine synthesis pathway (SSP) (Kottakis et al., 2016). While traditionally associated with epithelial cell adhesion, E-cadherin upregulates SSP in breast cancer, providing essential metabolic precursors for biosynthesis and oxidative stress resistance (Lee et al., 2024). This enables E-cadherin^+^ breast cancer cells to grow faster and metastasize more efficiently. Inhibiting PHGDH, specifically disrupts these processes, impairing cell proliferation and metastatic potential, making it a promising therapeutic target for E-cadherin^+^ breast cancers (Lee et al., 2024). Mutant p53 (mutp53), rather than wild-type p53, directly enhances the expression of all SSP enzymes in breast cancer cells, promoting *de novo* serine synthesis. This effect is more pronounced under conditions of low amino acid availability, thereby facilitating breast tumor cell proliferation. Additionally, mutp53 can promote glucose uptake, which also provides nutrients for serine synthesis via glycolysis. When glucose is scarce, mutp53 upregulates the expression of the gluconeogenesis enzyme PCK2, ensuring sufficient Ser/Gly production. This might enhance serine production by ensuring substrate availability and diverting carbon units from glycolysis to SSP (Tombari et al., 2023).

The overexpression of PHGDH and PSAT1 is significantly associated with poor clinical outcomes and malignant phenotypes in breast cancer. This overexpression can turn into a targetable metabolic vulnerability in specific contexts. For example, TNBC and HER2-positive breast cancer cells where isocitrate dehydrogenase 2 (IDH2) is highly expressed, the serine biosynthesis pathway becomes indispensable for sustaining their metabolic demands and aggressive phenotypes (Barnabas et al., 2021). IDH2 plays a key role in the TCA cycle, converting isocitrate to α-KG, and also drives the reductive carboxylation of α-KG to citrate (Fig. 2). Inhibiting PHGDH or PSAT1 in IDH2-high cells significantly disrupts the TCA cycle by reducing the availability of α-KG and other intermediates, thereby impairing mitochondrial respiration and energy production. Notably, PHGDH inhibitors effectively suppress the growth of IDH2-overexpressing breast tumors in pre-clinical models, indicating that targeting PHGDH could be a promising approach for TNBC with IDH2 overexpression (Barnabas et al., 2021). Moreover, PHGDH, as the first key enzyme in SSP, is overexpressed in both TNBC and BLBC, partly due to copy number amplification, and its catalytic activity is crucial for tumor proliferation (Possemato et al., 2011). The sustained ectopic expression of PHGDH in the breast epithelial cell line MCF10A disrupts acinar morphology and induces further phenotypic changes, leading to malignant transformation (Locasale and Cantley, 2011). It has also shown that the heterogeneity of PHGDH expression is closely related to the dissemination and metastatic potential of breast cancer cells. Approximately 67% of breast cancers exhibit homogeneously high PHGDH expression, while 33% show heterogeneous or low expression. Tumors with homogeneously high PHGDH expression tend to have higher tumor staging (pT), but interestingly, lymph node staging (pN) is higher in tumors with heterogeneous or low PHGDH expression (Rossi et al., 2022). When PHGDH is highly expressed in primary breast cancer, it can enhance tumor cell proliferation through its catalytic function; conversely, when expression is heterogeneous or low, it promotes integrin αvβ3 sialylation, thereby enhancing tumor metastasis and invasiveness (Rossi et al., 2022). Therefore, the presence of PHGDH heterogeneity in primary tumors can be considered a marker of tumor aggressiveness.

### Aspartate-asparagine metabolism

**Figure 3.**
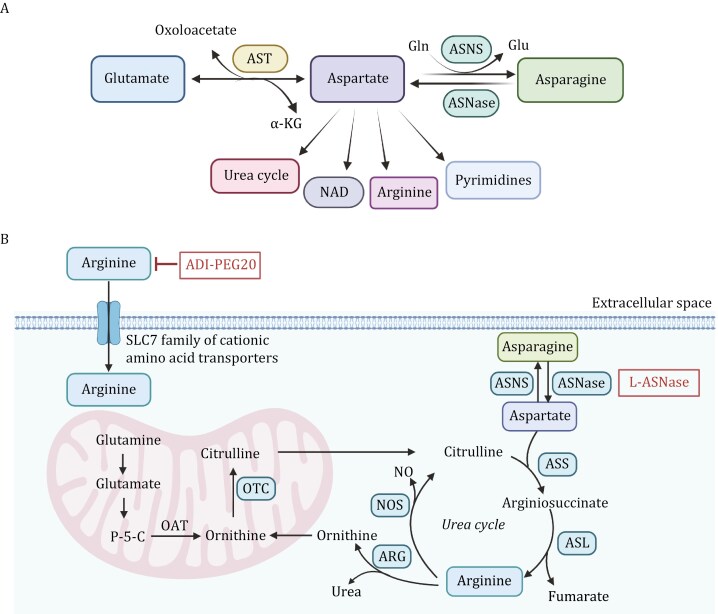
**Aspartate-asparagine metabolism and arginine metabolism pathways.** (A) Transamination of glutamate with oxaloacetate, reversible catalyzed by AST, produces aspartate and α-KG. Aspartate is a precursor for multiple pathways, including the synthesis of asparagine through ASNS using Gln as a nitrogen donor and its hydrolysis by ASNase to regenerate aspartate. Additionally, aspartate contributes to the urea cycle, NAD biosynthesis, arginine production, and pyrimidine nucleotide biosynthesis. (B) Asparagine is synthesized from aspartate through the action of ASNS. Arginine is imported into the cell and plays a crucial role in the urea cycle, which helps eliminate nitrogen waste through urea synthesis. Within the mitochondria, glutamine is converted to glutamate and further to P-5-C by the action of OAT. Citrulline reenters the cytoplasm, where it is converted back to arginine via ASS and ASL. NOS uses arginine to generate NO and ornithine, while ARG produces urea, completing the urea cycle. The inhibition of arginine via ADI-PEG20 and the degradation of asparagine via L-ASNase are depicted, demonstrating potential strategies to starve cancer cells of essential amino acids, thereby suppressing tumor growth. AST: aspartate transaminase; ASNS: asparagine synthetase; Gln: glutamine; ASNase: asparaginase; P-5-C: pyrroline-5-carboxylate; OAT: ornithine aminotransferase; ASS: argininosuccinate synthase; ASL: argininosuccinate lyase; NOS: Nitric oxide synthase; NO: nitric oxide; ARG: arginase. The figure was created with BioRender.com.

Aspartate is a non-essential amino acid in the body, and L-aspartate (L-Asp) is its predominant form. The growth and proliferation of most cells depend on the autonomously synthesized aspartate, which is primarily synthesized in the mitochondria (Garcia-Bermudez et al., 2018). Aspartate transaminase (AST) or aspartate aminotransferase, also known as AspAT/ASAT/AAT or (serum) glutamic oxaloacetic transaminase (GOT, SGOT) catalyzes the reversible transfer of an amino group between glutamate and oxaloacetate, producing aspartate and α-KG (Fig. 3A). Aspartate is then converted into asparagine by asparagine synthetase (ASNS), with the amino group provided by glutamine (Ling et al., 2023). Aspartate also participates in the biosynthesis of NAD through aspartate oxidase. Aspartate is also a crucial substrate for the *de novo* synthesis of pyrimidines and plays a significant role in oxidative phosphorylation (Rabinovich et al., 2015). Moreover, aspartate plays a role in the urea cycle, facilitating the clearance of ammonia from the body. Numerous studies have shown that L-Asp is essential for cell proliferation, and in cancer cells, most L-Asp is derived from mitochondrial glutamine metabolism, making it a limiting metabolite for cancer cell growth (Gorgoglione et al., 2022) (Fig. 3B). Besides its metabolic role, aspartate can serve as a signaling molecule that promotes metastasis aggressiveness. In metastatic breast cancer cells colonizing the lung, aspartate binds and activates the NMDA receptor to boost the translational regulation of collagen synthesis which is crucial for lung metastasis formation (Doglioni et al., 2025).

Notably, a study investigating plasma metabolic markers in breast cancer patients and healthy controls revealed a significant association between aspartate levels and the presence of breast cancer. The findings demonstrated that aspartate not only effectively distinguishes breast cancer patients from healthy controls but also enables differentiation between early-stage breast cancer (stages I–II) and healthy individuals. Moreover, the sensitivity and specificity of aspartate as a diagnostic marker for breast cancer were found to approach 100%, highlighting its potential as a highly accurate biomarker for early detection (Xie et al., 2015). In breast cancer tissues, the levels of aspartate and its related metabolites (asparagine and nucleosides) were significantly higher than in non-tumor tissues. Breast cancer cells also showed a significantly higher level of aspartate metabolism compared to normal breast cells, and the activity of GOT was notably altered (Xie et al., 2015). These findings suggest that the metabolic needs of breast tumor cells require the absorption of aspartate from the bloodstream, leading to a decrease in serum aspartate levels and an increase in aspartate levels in tumor tissues as a critical metabolic feature of breast cancer. Additionally, the levels of aspartate are regulated by asparagine synthetase. Overexpression of insulin-like growth factors 1 and 2 (key regulators of breast cancer development) in breast cancer tissues leads to increased expression of asparagine synthetase, which, in turn, alters the levels of asparagine and aspartate (Xie et al., 2015). Furthermore, the loss of p53 can upregulate the expression of ASNS, affecting the balance of asparagine and aspartate both inside and outside the tumor cells, thereby sustaining the survival and proliferation capacity of the tumor cells. Removing asparagine can significantly inhibit tumor cell proliferation and induce apoptosis (Deng et al., 2020).

### Arginine metabolism

Arginine plays a crucial role in various cellular biological processes, including cell proliferation, cell signaling, and immunity. Arginine in cells can be replenished through dietary intake rich in arginine or synthesized *de novo* from citrulline and aspartate (Wei et al., 2020). This synthesis occurs through a two-step catalysis by argininosuccinate synthase 1 (ASS1) and argininosuccinate lyase (ASL) (Fig. 3B). Arginase 1 (Arg1) then breaks down arginine into ornithine and urea (Sun and Zhao, 2022). The enzyme ornithine transcarbamylase (OTC) converts ornithine back into citrulline in the mitochondria, allowing for recycling (Sun and Zhao, 2022). Abnormalities in ASS1, ASL, or OTC can affect intracellular arginine storage. Therefore, arginine is also considered a “semi-essential” or “conditionally essential” amino acid. Normal cells do not completely depend on external arginine. However, in many cancer cells, including breast cancer, the transcription of ASS1 is suppressed, causing these cells to lose the ability to synthesize arginine (Qiu et al., 2014). As a result, the cells become addicted to external arginine, a phenomenon referred to as arginine auxotrophic cells.

More than 60% of breast cancer biosamples show low or absent ASS1 levels. This low ASS1 abundance is significantly correlated with lower survival rates in breast cancer (Qiu et al., 2014). Arginine starvation in arginine auxotrophic breast cancer cells induces ASNS, depletes aspartate in these tumor cells, and disrupts their malate-aspartate shuttle. The fate of arginine-starved cells is influenced by mitochondrial dysfunction and intracellular aspartate depletion. Supplementing aspartate, inhibiting ASNS, or depleting mitochondria in these breast cancer cells can provide protection. Additionally, in a breast cancer xenograft mouse model, dietary arginine restriction reduced the growth of ASS1-deficient breast cancer tumors in mice (Sullivan et al., 2018). This suggests that breast cancer patients with ASS1 deficiency might be candidates for arginine starvation therapy. Furthermore, in TNBC cells, arginine depletion can inhibit the translation of the typical endoplasmic reticulum (ER) stress marker, binding immunoglobulin protein (BiP), through ribosomal stalling. This triggers an atypical ER stress response that disrupts protein homeostasis and plays a crucial role in inhibiting TNBC cell growth, indicating that BiP acts as a protective agent in arginine depletion-induced growth inhibition (Vidal et al., 2023).

Arginine can be metabolized into nitric oxide (NO) and citrulline by nitric oxide synthase (NOS) and into ornithine and urea by arginase (ARG). Breast cancer cells rely on ARG2 for ornithine synthesis, while normal cells use ornithine aminotransferase (OAT). Knocking down ARG2 in breast cancer cells significantly inhibits cell proliferation and causes G_2_/M arrest, without compensation through the OAT pathway (Roci et al., 2019). Similarly, knocking out ASL in TNBC cells leads to a delayed G_2_/M transition. NO produced by NOS from arginine has a dual role in cancer signaling. On one hand, NO is considered an anti-tumor agent due to its antioxidant capacity and free radical scavenging properties. On the other hand, increasing evidence suggests that NO promotes tumor angiogenesis, metastasis, and anti-apoptotic processes (Chen et al., 2021; Keshet and Erez, 2018). Elevated NO production can induce S-nitrosylation of EGFR, thereby activating various oncogenic signaling pathways, including c-Myc, Akt, and mTOR (Switzer et al., 2012). Additionally, NO can directly inhibit the catalytic activity of the demethylase KDM3A, thereby altering the histone methylation pattern and regulating the expression levels of various oncogenes (Hickok et al., 2013).

### Tryptophan metabolism

**Figure 4.**
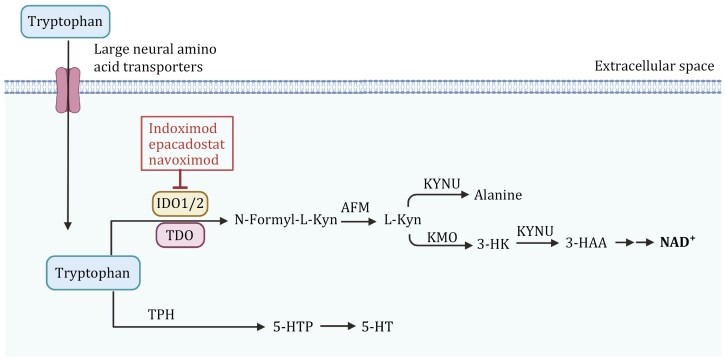
**Tryptophan metabolism pathway and its regulation in cancer cells.** The enzyme indoleamine 2,3-dioxygenase (IDO1/2) and tryptophan 2,3-dioxygenase (TDO) convert tryptophan into N-formyl-L-kynurenine, which is further metabolized into L-kynurenine (L-Kyn) by arylformamidase (AFM). L-Kyn undergoes multiple conversions via kynureninase (KYNU) to alanine, and through kynurenine monooxygenase (KMO) and KYNU into 3-hydroxykynurenine (3-HK) and 3-hydroxyanthranilic acid (3-HAA), leading to the production of NAD^+^, a key molecule in cellular energy metabolism. Tryptophan is also converted into serotonin (5-HT) through the intermediate 5-hydroxytryptophan (5-HTP) by tryptophan hydroxylase (TPH). Tryptophan degradation through IDO1/2 can be inhibited by several drugs, including Indoximod, Epacadostat, and Navoximod, which are shown to block the immunosuppressive effects of kynurenine accumulation in cancer cells. The figure was created with BioRender.com.

Tryptophan is an essential amino acid in the human body and must be obtained from the diet to maintain cell function. The metabolic pathways of tryptophan in the body are relatively complex and mainly include the following three processes: (i) the production of indole and its derivatives; (ii) the formation of serotonin (5-hydroxytryptamine (5-HT)) involving tryptophan hydroxylase 1 (TPH); and (iii) more than 95% of free Trp is catalyzed by three rate-limiting enzymes, indoleamine 2,3-dioxygenase 1 (IDO1), IDO2, and tryptophan 2,3-dioxygenase 2 (TDO2) to produce kynurenine (Xue et al., 2023) (Fig. 4). In the human body, enzymes related to tryptophan metabolism exhibit tissue specificity, and their expression is tightly regulated. For example, IDO1 is expressed in various tissues throughout the body, while TDO2 is primarily expressed in the liver. The bioactive metabolite kynurenine, produced by these enzymes, may affect cancer development by modulating immune surveillance or oncogenic signaling (Platten et al., 2019).

Compared to healthy controls, breast cancer patients exhibit significantly lower levels of kynurenine, tryptophan, and their ratio in plasma, suggesting an increased breakdown of tryptophan in these patients. In hormone receptor-negative breast cancer patients, the plasma tryptophan levels are lower, and the kynurenine/tryptophan ratio is higher compared to hormone receptor-positive cancers, while this ratio is lower in lobular carcinoma than in any other histological subtype (Onesti et al., 2019). The expression of TDO2 and IDO1 is upregulated in breast cancer, with high levels of TDO2 closely linked to poor patient prognosis (D’Amato et al., 2015; Jacquemier et al., 2012). Moreover, elevated TDO2 expression in clinical samples of TNBC correlates with higher disease grades, estrogen receptor-negative status, and reduced overall survival. In TNBC cells, TDO2 is overexpressed in a nuclear factor kappa-light-chain-enhancer of activated B cells (NF-κB) dependent manner, activating the endogenous kynurenine receptor aryl hydrocarbon receptor (AhR) by increasing kynurenine production. Pharmacological inhibition or genetic suppression of TDO2 or AhR enhances the sensitivity of TNBC cells to apoptosis and reduces the proliferation, migration, and invasion of TNBC cells. In a mouse model of breast cancer with TNBC cells treated with a TDO2 inhibitor, there was a significant reduction in the number of metastatic lung nodules, indicating that TDO2 enhances the metastatic capacity of TNBC cells (D’Amato et al., 2015). Interestingly, kynurenine and xanthurenic acid produced by the TDO-dependent kynurenine pathway can drive AhR activity in TNBC cells. In turn, activated AhR can promote TDO expression to form a positive feedback loop and promote the migration of breast cancer cells (Novikov et al., 2016). IDO1 regulates the cell cycle distribution and exhibits anti-apoptotic effects in breast cancer cells. IDO1-positive breast tumors in immunocompetent mice show significantly higher levels of spontaneous lung metastasis compared to IDO1-negative tumors, while the opposite was observed in immunodeficient mice, confirming the immunosuppressive role of IDO1 in spontaneous metastasis formation (Levina et al., 2012).

In addition, IDO1 is also upregulated in cancer-associated immune cells, such as dendritic cells and macrophages, which promotes immune evasion by depleting tryptophan in the tumor microenvironment, particularly at the immunological synapse (Meireson et al., 2020). Tryptophan depletion activates the GCN2 stress response in cytotoxic T cells, leading to reduced proliferation, impaired cytokine production, functional exhaustion, and susceptibility to apoptosis. Additionally, regulatory T cells (Tregs) are promoted, further enhancing immune tolerance (Sarangi, 2024). Kynurenine reinforces the immunosuppressive phenotype by activating AhR on T cells. Together, these mechanisms inhibit effective anti-tumor immunity, enabling tumors to evade immune surveillance.

### Other amino acids

Breast cancer cells can become dependent on exogenous methionine, accelerating tumor proliferation. Methionine restriction inhibits the progression of breast cancer in mice by reducing cell proliferation and increasing apoptosis (Strekalova et al., 2015). Leucine, isoleucine, and valine as branched-chain amino acids (BCAAs) can activate mTORC1 signaling, which stimulates protein translation, growth, and survival, and involved in breast cancer progression (Jung et al., 2021). Inhibiting the breakdown of BCAAs can suppress breast cancer cell migration, invasion, and N-cadherin expression. Additionally, it can inhibit tumor growth and lung metastasis in mice (Chi et al., 2022).

### The role of mitochondria in breast cancer

Mitochondria play a central role in cancer metabolism, serving as metabolic hubs for energy production, biosynthesis, and redox homeostasis. In breast cancer, mitochondrial functions are closely linked to amino acid metabolism, contributing to tumor growth and adaptation under stress conditions. For instance, glutaminolysis, a key mitochondrial metabolic pathway, converts glutamine-derived glutamate into α-KG, which fuels the TCA cycle (Wang et al., 2020b). This process provides intermediates necessary for energy production, biosynthetic precursors, and redox balance to support the rapid proliferation of cancer cells. Additionally, mitochondrial-dependent tryptophan metabolism generates metabolites, such as kynurenic acid, which suppress anti-tumor immune responses (Liu et al., 2024b; Triplett et al., 2018). Similarly, mitochondrial utilization of arginine-derived intermediates supports anabolic pathways, including nucleotide, protein, and polyamine synthesis, while promoting metabolic vulnerabilities in arginine-auxotrophic tumors (Liu et al., 2024a).

Beyond metabolic roles, mitochondria are essential for maintaining bioenergetics in breast cancer cells. Under conditions of metabolic stress, such as hypoxia or nutrient deprivation, oxidative phosphorylation (OXPHOS) becomes critical for ATP production and survival, particularly in TNBC (El-Botty et al., 2023). Moreover, mitochondrial dynamics, including fusion, fission, and biogenesis, enhance metabolic plasticity, enabling cancer cells to adapt to stress, resist apoptosis, and promote metastasis (Hsu et al., 2024). Dysregulation of these processes can drive metastatic progression and therapeutic resistance. Future research integrating mitochondrial biology with precision medicine approaches could pave the way for novel therapies targeting metabolic vulnerabilities in breast cancer.

## Clinical application of amino acid metabolic reprogramming in breast cancer

### Clinical diagnostic imaging

Currently, the clinical application of ^18^F-fluorodeoxyglucose positron emission tomography (FDG-PET) technology has been widely used for whole-body staging of malignant tumors, detection of recurrent diseases, and monitoring of treatment response. ^18^F-FDG is mainly based on the avid uptake of glucose by tumors, making it a tumor tracer. However, the main limitation of ^18^F-FDG-PET is that some inflammatory diseases and benign tumors also have a high uptake rate of ^18^F-FDG (Alcin et al., 2023). Due to amino acid reprogramming in breast cancer cells, the use of natural or synthetic amino acid radiotracers for molecular imaging holds promising potential for application.

Anti-1-amino-3-^18^F-fluorocyclobutane-1-carboxylic acid (^18^F-FACBC) is a synthetic L-leucine analog mainly transported by SLC7A5 and SLC1A5. The uptake of anti-^18^F-FACBC is not only associated with the malignancy of breast tumor cells but also shows a high uptake rate in an *in situ* breast cancer mouse model (Liang et al., 2011). ^18^F-Fluciclovine PET/CT imaging in patients with breast lesions indicates that the uptake in breast cancer is significantly higher than in benign lesions and normal breast tissue. It may also be used to detect unsuspected axillary lymph node metastases in breast cancer, including micrometastases (≤2 mm) in lymph node lesions (Tade et al., 2016).

(4S)-4-(3-^18^F-fluoropropyl)-L-glutamic acid (BAY 94-9392, also known as ^18^F-FSPG) is a glutamate derivative specifically transported by the xC-transporter. It has demonstrated excellent tumor visualization in preclinical animal models. However, in a study imaging five breast cancer patients using ^18^F-FSPG PET, three cases could be visually identified, while two cases of breast cancer lesions over 7 cm were missed but could be detected by conventional imaging methods (Baek et al., 2012).

Leveraging the characteristic glutamine addiction of cancer cells, a natural glutamine analog, ^18^F-(2S,4R)4-fluoroglutamine, has been developed as a potential tumor imaging tracer. ^18^F-FGln PET can be used to track the size of the glutamine pool in breast cancer cells with different GLS activity levels, serving as a reverse indicator of glutamine catabolism (Zhou et al., 2017). The peak SUVmax of ^18^F-FGln in breast cancer patients is reached within 10 min, and tumors and metastatic lymph nodes in nearly all of the 17 tested breast cancer patients could be diagnosed using ^18^F-FGln imaging (Xu et al., 2020). Using ^99m^Tc-labeled DTPA-bis-methionine (DTPA-bis-MET) for scintigraphy in breast cancer detection showed a 96% sensitivity and positive predictive value and a 67.0% specificity (Sharma et al., 2014).

### Targeting amino acid transporters

Solute carriers (SLCs) are a group of membrane transport proteins. To date, the SLC superfamily has been identified to consist of over 460 transporter proteins classified into 66 distinct families, and about 20% of the SLC superfamily is mainly used to transport neutral amino acids such as glutamine. Rapidly proliferating cancer cells often need to upregulate the expression of amino acid transporter proteins to obtain abundant amino acids from the extracellular environment in order to meet their needs. Dysregulation of amino acid transporter proteins leads to metabolic reprogramming of tumors, and different subtypes of breast cancer exhibit specific amino acid transporter protein profiles. It has been demonstrated that SLC1A5, SLC6A14, SLC7A11, SLC3A1, SLC7A5, SLC38A2, and SLC38A5 are significantly expressed in breast cancer cells (Table 1).

**Table 1.** Targeting amino acid metabolism and potential personalized medicine application in breast cancer. A comprehensive overview of amino acid metabolism-related targets, molecular phenotypes, and their diagnostic and therapeutic potentials in breast cancer. It also includes inhibitors and their current statuses, offering insight into personalized medicine applications.

			Potential personalized medicine application					
Function classification	Target	Amino acid	Molecular phenotype	Diagnostic potential	Therapeutic potential	Inhibitor	Status	Reference
Amino acid transporters	SLC1A5	Glutamine	Overexpression in HER2+ and TNBC	Overexpression linked to poor prognosis in HER2+ and TNBC; Potential biomarker for metabolic dependency	Enables stratification of patients with HER2+ and TNBC for therapies targeting glutamine metabolism	GPNA	Preclinical tool (*in vitro*)	Reynolds et al., 2014; Broer et al., 2018; Schulte et al., 2018; Zhou et al., 2016
						Benzylserine	*In vitro*	
						V-9302	*In vitro* and in PDX nude mice	
						1,25(OH)_2_D	*In vitro*	
	SLC6A14	All neutral amino acids; All cationic amino acids	Elevated in ER+	Biomarker for aggressive disease in ER+ breast cancer	Stratifies ER+ patients for therapies targeting neutral amino acid uptake and metabolism	α-MT	*In vitro* and in spontaneous mouse models	Babu et al., 2015; Karunakaran et al., 2011
	SLC7A11	Cystine, glutamate	High SLC7A11 in ER−, claudin low, and TNBC	Biomarker for ferroptosis resistance and metabolic vulnerabilities	Exploits SLC7A11 overexpression for inducing ferroptosis in resistant BC	Anti-xCT vaccination	Preclinical tool (*in vitro*)	Timmerman et al., 2013; Yang et al., 2014; Ruiu et al., 2019; Nath et al., 2024; Yang et al., 2021a
						Metformin	Preclinical tool (*in vitro*)	
						Erastin	Mammary fat pad Tool compound, induces iron-dependent ferroptosis	
						Niclosamide	Preclinical tool (*in vitro* and *in vivo*)	
	SLC7A5	Leucine, others	Overexpression in HER2+, ER+ and TNBC	Associated with metastatic potential and recurrence	Targets leucine dependency in aggressive BC	BCH	Preclinical tool compound (*in vitro*)	El Ansari et al., 2018; Huang et al., 2023; Liang et al., 2011; Huang et al., 2023
						JPH203	Preclinical tool compound (*in vivo*)	
	SLC38A2	Neutral amino acids	Overexpression in TNBC	Overexpression may serve as a diagnostic marker for TNBC metabolic activity	Stratification of TNBC for therapies targeting neutral amino acid uptake	MeAIB	Preclinical tool compound (*in vitro*)	Ruan et al., 2024; Morotti et al., 2019; Gauthier-Coles et al., 2022
						MMTC	Preclinical tool compound (*in vitro*)	
	SLC38A5	Neutral amino acids	Overexpression in TNBC	May be linked to metabolic adaptations in TNBC	Therapy to target H^+^ antiport activity in TNBC metabolic reprogramming	Niclosamide	Preclinical tool (*in vitro* and *in vivo*)	Ramachandran et al., 2021; Sennoune et al., 2023; Mathew et al., 2024
Amino acid metabolizing enzymes	GLS	glutamine	GLS1 (TNBC); GLS2 (ER+)	Potential marker for stratifying patients based on glutamine metabolism dependency	GLS1 targeting for TNBC; GLS2 targeting for ER+ subtypes	BPTES	Preclinical tool compound (*in vitro*)	Robinson et al., 2007; Jeong et al., 2013; Chen et al., 2016; Lukey et al., 2019; Wicker et al., 2021
						Compound 968	Preclinical tool compound (*in vitro*)	
						CB-839	Phase I/II clinical trials	
	PHGDH		High PHGDH expression in TNBC	Elevated PHGDH expression linked to serine biosynthesis in TNBC	Targets serine synthesis pathway in serine-addicted TNBC	NCT-503	Preclinical tool (*in vitro* and *in vivo*)	Pacold et al., 2016; Mullarky et al., 2016; Wang et al., 2017; Tajan et al., 2021
						CBR-5884	Preclinical tool (*in vitro*)	
						PKUMDL-WQ-2101	Preclinical tool (*in vitro* and *in vivo*)	
	Asparagine	Asparagine	Dependency in asparagine auxotrophic tumors.	May serve as a biomarker for tumors reliant on asparagine metabolism	Therapy to target asparagine metabolism in highly auxotrophic tumors	L-ASNase	Preclinical tool compound (*in vitro*)	Brumano et al., 2018; Shahnazari et al., 2022
	Arginine	Arginine	Low ASS1 expression in certain BC subtypes	Biomarker for stratifying patients with arginine auxotrophy	Targeting ASS1-deficient tumors with arginine deprivation therapies	ADI-PEG20	Phase I clinical trial	Yang et al., 2010; Yao et al., 2022
	IDO1	Tryptophan	-	Potentially linked to immune evasion in breast cancer microenvironment.	Targeting tryptophan metabolism to restore immune responses	Indoximod	Phase II clinical trial	Guney Eskiler and Bilir, 2021; Mariotti et al., 2021; Hong et al., 2018; Nayak-Kapoor et al., 2018; Jung et al., 2019
						Epacadostat	Phase I/II clinical trials	
						Navoximod	Phase I clinical trial	

#### SLC1A5

SLC1A5, also known as ASCT2, is a cell surface sodium-dependent neutral amino acid exchanger. It is one of the most studied glutamine transporters and is closely related to the progression of many malignant solid tumors, including breast cancer (Cormerais et al., 2018; Wang et al., 2020a). In addition to controlling glutamine uptake, SLC1A5 can also transport other amino acids, such as alanine, serine, cysteine, threonine, and asparagine. SLC1A5 is significantly overexpressed in most subtypes of breast cancer, but different breast cancer cell lines show varying degrees of dependence on SLC1A5 activity. When treated with the SLC1A5 inhibitor L-γ-glutamyl-p-nitroanilide (GPNA), different molecular subtypes of breast cancer cells, including luminal, basal-like, and claudin-low, showed significant inhibition of glutamine uptake (van Geldermalsen et al., 2016). However, GPNA treatment specifically inhibited the growth and cell cycle progression of basal-like cells. Furthermore, knockdown of SLC1A5 induced rapid cell death in basal-like triple-negative breast cancer cells *in vitro* and reduced xenograft implantation and subsequent growth in mice but had little effect on luminal cells (van Geldermalsen et al., 2016). This indicates that SLC1A5 is crucial for the growth and progression of triple-negative breast cancer, with less impact on luminal breast cancer.

However, an *in vitro* study suggested that SLC1A5 might be associated with endocrine therapy failure in some luminal breast cancers. They found that SLC1A5 was upregulated in endocrine therapy-resistant breast cancer cells, and inhibition of SLC1A5 suppressed the proliferation of breast cancer cells resistant to aromatase inhibitors (Chen et al., 2015). Clinical sample analysis suggested that luminal breast cancer patients with high SLC1A5 expression are more likely to experience relapse after endocrine treatment. Knockdown of SLC1A5 increased the sensitivity of luminal breast cancer cells to tamoxifen treatment, possibly by modulating the expression of the pentose phosphate pathway enzyme TALDO1, which is crucial for cancer cell growth and proliferation. Therefore, SLC1A5 and TALDO1 can be used to predict the prognosis of endocrine therapy in luminal breast cancer (Alfarsi et al., 2021).

SLC1A5 is associated with the transcriptional regulators MYC and ATF4 in TNBC, and the transcriptional programs driven by MYC and ATF4 can dynamically regulate SLC1A5 (van Geldermalsen et al., 2016). The tumor suppressor gene Rb loss can lead to the activation of its regulated transcription factor E2F-3, which directly regulates SLC1A5 mRNA and protein expression to promote glutamine uptake (Reynolds et al., 2014). Tumor suppressor miR-137 belongs to a class of small non-coding RNAs that block translation and promote degradation of SLC1A5 mRNA by targeting SLC1A5 mRNA, resulting in increased SLC1A5 expression and enhanced glutamine metabolism (Dong et al., 2017). The multidrug resistance-associated gene ABC transporter ABCG2 can form a complex with SLC1A5 in MCF-7 cells to facilitate glutamine influx and enhance redox regulation, providing a survival advantage to cancer cells under oxidative stress (Shi et al., 2024).

Targeting SLC1A5 to prevent extracellular glutamine uptake and metabolism represents a promising direction for breast cancer therapy. Benzylserine was the first molecule discovered to inhibit SLC1A5 in breast cancer cells, disrupting intracellular glutamine uptake. However, it was found to have four additional targets, making it nonspecific (van Geldermalsen et al., 2018). GPNA can inhibit the proliferation of TNBC cells, but glutamine analogs can block multiple glutamine transporters such as SNAT1 and SNAT2. Due to its lack of specificity, low affinity, and toxicity, GPNA is not suitable for clinical use (Broer et al., 2018). The small molecule lead compound V-9302, derived from 2-amino-4-bis (aryl oxybenzyl) amino butyric acid, is one of the most effective inhibitors of active cellular glutamine uptake. It inhibits SLC1A5-mediated glutamine uptake in human cells in a concentration-dependent manner, with potency increased 100-fold compared to GPNA. V-9302 can reduce breast cancer cell growth and proliferation, increase cell death, and enhance oxidative stress, effectively promoting anti-tumor responses both *in vitro* and in mouse models (Schulte et al., 2018).

The oncogene ras is mutated in various tumor types, and Harvey-ras transformed MCF10A human breast epithelial cells (MCF10A-ras) can serve as a cell model for early breast cancer progression. A functional negative vitamin D response element exists in the *SLC1A5* gene promoter. The active form of vitamin D, 1,25(OH)₂D, can decrease SLC1A5 transcript levels and inhibit glutamine uptake in MCF10A-ras cells (Zhou et al., 2016). MEDI7247, a novel pyrrolobenzodiazepine (PBD) antibody-drug conjugate targeting SLC1A5, specifically binds to surface SLC1A5. It is currently in Phase I clinical trials for relapsed/refractory hematologic malignancies. However, adverse events related to MEDI7247 have hindered repeated dosing and response durability, leading to premature termination of the study (Maris et al., 2024; Wichmann et al., 2023). Although inhibition of SLC1A5 has shown some anti-tumor effects *in vitro* and in mouse models, safe and effective specific inhibitory drugs are still lacking and require further development.

#### SLC6A14

SLC6A14, also known as amino acid transporter B^0,+^ (ATB^0,+^), mediates the unidirectional influx of amino acids. This transport is driven by the transmembrane gradients of Na^+^ and Cl^−^ and is coupled with the membrane potential (Nalecz, 2020). SLC6A14 has a broad substrate specificity, capable of transporting all amino acids except the acidic ones, aspartate and glutamate. Studies have found that the expression of SLC6A14 is specifically upregulated in ER-positive breast cancer tissues and cell lines (Karunakaran et al., 2011). This upregulation likely supports the increased demand for amino acids, such as leucine, glutamine, and arginine. Furthermore, upon treatment with estradiol, which uses luciferase as a reporter gene, an ER binding site was identified in the *SLC6A14* promoter in ER-positive breast cancer cells (Karunakaran et al., 2011). Estradiol treatment enhances SLC6A14 expression in ER-positive cell lines, and this effect can be reversed by antiestrogen therapy using tamoxifen (Karunakaran et al., 2011). In spontaneous mouse models of breast cancer, enhanced SLC6A14 expression promotes the growth of ER-positive breast tumors, while *SLC6A14* gene knockout mice exhibit breast tumors with amino acid starvation, weakened mTOR signaling, and reduced cell proliferation (Babu et al., 2015). Although endocrine therapy is effective for ER-positive breast cancer patients and is the standard treatment, approximately 40% of female patients experience cancer recurrence due to endocrine therapy resistance. Global transcriptomic analysis of endocrine therapy-resistant breast cancer cells revealed that SLC6A14 expression is downregulated under the control of increased miR-23b-3p expression, resulting in increased aspartate and glutamate uptake (Bacci et al., 2019).

The selective blocker of SLC6A14, α-methyl-dl-tryptophan (α-MT), when used *in vitro* on ER-positive MCF7 breast cancer cells, can induce amino acid deprivation, inhibit mTOR, and activate autophagy. Prolonged treatment with α-MT leads to apoptosis, and adding an autophagy inhibitor also induces apoptosis in aiSLC6A14-dependent manner. In mouse xenograft models, α-MT treatment effectively reduced the tumor growth of ER-positive breast tumors but does not affect the tumor growth of ER-negative breast cancer cells (Karunakaran et al., 2011).

SLC6A14 must leave the endoplasmic reticulum and reach the plasma membrane to function as a transporter, a step mediated by heat shock proteins HSP70 and HSP90beta (Nalecz, 2020). Currently, ganetespib (an HSP90 inhibitor) has been reported to be used in combination with various chemotherapeutic drugs in phase II clinical trials for treating ER-positive and HER2-positive metastatic breast cancer patients (NCT01560416, NCT02060253), with encouraging results in HER2+ metastatic breast cancer. Therefore, besides directly inhibiting SLC6A14 itself, interfering with the transport of SLC6A14 by inhibiting HSP appears to be a promising strategy (Jhaveri and Modi, 2015).

#### SLC7A11

SLC7A11, also known as xCT, is a chloride ion-dependent, sodium-independent cystine-glutamate antiporter located on the cell surface. SLC7A11 facilitates the exchange of intracellular glutamate and extracellular cystine in a 1:1 ratio. Once inside the cell, cystine is converted to cysteine through a reduction reaction that consumes NADPH. This cysteine is subsequently used for the biosynthesis of glutathione (Conrad and Sato, 2012). The single-pass transmembrane protein SLC3A2 forms a heterodimer with SLC7A11 via a disulfide bond, serving as a chaperone protein that maintains the stability and proper membrane localization of SLC7A11 (Koppula et al., 2018). SLC7A11 is upregulated in breast cancer, particularly in those with poor prognosis, such as ER-negative, claudin-low, and TNBC, which show higher levels of SLC7A11 mRNA and protein expression. This expression is closely associated with poor patient outcomes (Timmerman et al., 2013). *SLC7A11* gene expression is positively correlated with other amino acid transporters like SLC3A2, SLC1A5, GLS, and the infiltration of neutrophils and macrophages, suggesting that SLC7A11 plays a coordinated role in metabolic regulation and tumor immunity (Nath et al., 2024). The high uptake of cystine mediated by SLC7A11 in cancer cells, coupled with glutamate export, makes SLC7A11-high cancer cells heavily reliant on glutamine and glucose (Timmerman et al., 2013). Furthermore, the influx of cystine via SLC7A11 promotes GSH biosynthesis, which in turn aids in the detoxification of lipid peroxides mediated by GPX4, thereby inhibiting ferroptosis, a form of iron-dependent cell death (Yang et al., 2014).

The proteasome chaperone Gankyrin, which is highly expressed in TNBC tissues and cells, can form a complex with MDM2 and p53, leading to the ubiquitination of p53. This increases the expression of SLC7A11, resulting in impaired cysteine uptake and reduced GPX4 production, thereby enhancing resistance to ferroptosis (Lei et al., 2023). Studies have shown that SLC7A11 plays a crucial role in the biology of breast tumor stem cells, leading to the design of various anti-SLC7A11 vaccine formulations, including DNA plasmid vaccines, virus-like particles (VLP), and bovine herpesvirus type 4 (BoHV-4) vaccines (Ruiu et al., 2019). In a breast cancer model established subcutaneously in BALB/c female mice, vaccination with anti-SLC7A11 formulations not only inhibited the growth kinetics of primary tumors but also reduced lung colonization. Additionally, metformin can reduce the protein stability of SLC7A11 in breast cancer cells by inhibiting its UFMylation, leading to increased intracellular Fe^2+^ and lipid ROS levels, thereby inducing ferroptosis (Yang et al., 2021a). The development of specific inhibitors targeting SLC7A11 is still inadequate and requires further efforts.

#### SLC7A5

SLC7A5, also known as the neutral amino acid transporter 1 (LAT1), is a sodium-independent transporter that mediates the efflux of intracellular glutamine to exchange for large neutral amino acids such as leucine. SLC7A5 exhibits notable subtype specificity in breast cancer, with high expression in HER2+, ER+, and TNBC, and is associated with poor patient prognosis (El Ansari et al., 2018). Gene expression analysis of 80 breast cancer tissues revealed that SLC7A5 expression is positively correlated with the expression of the proliferation marker Ki-67 and the hypoxia-inducible factor Hif1α, indicating a connection between SLC7A5 expression, breast cancer cell proliferation, and tissue hypoxia (Tornroos et al., 2022). In MCF-7 cells, SLC7A5 expression is significantly upregulated, and overexpression of SLC7A5 can increase the expression of phosphorylated AKT, phosphorylated mTOR, and phosphorylated p70-S6K, promoting the proliferation and cell cycle regulation of MCF-7 cells (Li et al., 2021). Activation of the AKT/mTORC1 pathway is crucial for the cell proliferation process and prevents tumor cell apoptosis. *In vivo* lung cancer metastasis model established in BALB/c mice with orthotopic breast tumors, knocking down SLC7A5 led to a reduction in tumor weight and volume, inhibited lung tumor lesion formation, and reduced the abundance of CD4^+^ T and CD8^+^ T cells, indicating that inhibiting SLC7A5 alleviated immune suppression (Huang et al., 2023). Furthermore, SLC7A5 plays a crucial role in endocrine therapy resistance in ER+ patients. LLGL2, a scaffold protein, is overexpressed in ER+ breast cancer and promotes ER+ breast cancer cell proliferation by regulating leucine uptake (Saito et al., 2019). Additionally, studies have found that the estrogen receptor targets LLGL2 expression, and LLGL2 interacts with SLC7A5 and the membrane fusion regulator YKT6 to form a trimeric complex to regulate the cell surface levels of SLC7A5 (Saito et al., 2019). The high expression of SLC7A5 promotes the proliferation of ER+ breast cancer cells, leucine absorption, and the induction of tamoxifen resistance, making SLC7A5 a necessary and sufficient condition for developing resistance to tamoxifen therapy.

2-Amino-bicyclo[2.2.1]heptane-2-carboxylic acid (BCH) is a nonspecific and concentration-dependent inhibitor of L-type amino acid transporters, which does not selectively inhibit SLC7A5. The addition of BCH to breast cancer cells can effectively suppress their proliferation and colony formation (Liang et al., 2011). Triiodothyronine (T3), a thyroid hormone, serves as a substrate for SLC7A5, and researchers have designed a tyrosine analog based on the structure of T3 (KYT-0353 or JPH203) that can selectively inhibit SLC7A5 (Oda et al., 2010). In a study, mice with 4T1 subcutaneous fat pad tumors were randomly divided into four groups and injected with a vehicle, anti-PD-1 antibody, JPH203, or a combination of the two. The results showed that both the JPH203 group and the anti-PD-1 group were able to inhibit the growth and lung metastasis of TNBC, with the combined treatment group showing superior tumor suppression compared to the single-agent or control groups (Huang et al., 2023). However, further clinical trials are needed to potentially advance the clinical application of targeted metabolic therapy.

#### SLC38A2

The sodium-coupled neutral amino acid transporter, SLC38A2/SNAT2 mediates cellular uptake of glutamine and other small, neutral amino acids. SLC38A2 has been identified as a highly expressed amino acid transporter in six breast cancer cell lines, and high levels of SLC38A2 are associated with poor clinical outcomes in TNBC patients. Knockdown of SLC38A2 in breast cancer cells regulates mTORC1 signaling, reduces glutamine consumption in breast cancer cell lines, sensitizes cells to low-glutamine conditions, and increases ROS production (Morotti et al., 2021). Compared to patients with metastases to other organs, breast cancer patients with brain metastases have higher levels of miR-199b-5p in their blood. Extracellular vesicles from breast cancer cells with high brain metastatic potential carry elevated levels of miR-199b-5p. Breast cancer cell-secreted miR-199b-5p targets SLC1A2 in astrocytes and SLC38A2 and SLC16A7 in neurons, hijacking neuron-astrocyte metabolic coupling (Ruan et al., 2024). This leads to extracellular retention of glutamate, glutamine, and lactate, thereby promoting brain metastasis. SLC38A2 is also linked to drug resistance. Paclitaxel-induced endoplasmic reticulum stress in breast cancer cells promotes ubiquitination and degradation of SLC38A2 by the ubiquitin ligase RNF5, leading to increased autophagy and cell death. Conversely, increased SLC38A2 expression promotes tumorigenesis and reduces paclitaxel responsiveness (Jeon et al., 2015). Additionally, both *in vitro* and in mouse xenograft experiments, hypoxia upregulates SLC38A2 expression in an HIF-1α-dependent manner. Overexpression of SLC38A2 leads to complete resistance to anti-estrogen therapy and is induced in tamoxifen resistance, contributing to endocrine resistance in breast cancer (Morotti et al., 2019).

Pharmacological inhibition of SLC38A2, using system A inhibitors such as methylamino-isobutyric acid (MeAIB), has shown promising results in preclinical studies, reducing amino acid uptake and tumor cell viability. 3-(*N*-methyl (4-methylphenyl)sulfonamido)-*N*-(2-trifluoromethylbenzyl)thiophene-2-carboxamide (MMTC or 57E) has been identified as a potent inhibitor of SLC38A2 by FLIPR membrane potential (FMP) assay (Gauthier-Coles et al., 2022). These findings underscore the therapeutic potential of targeting SLC38A2 in breast cancer, particularly in aggressive subtypes and treatment-resistant cases.

#### SLC38A5

SLC38A5 is an amino acid-coupled Na^+^/H^+^ exchanger that mediates the influx of glutamine, glycine, serine, and methionine into cells in a Na^+^-dependent manner while exchanging intracellular H^+^, leading to intracellular alkalization. SLC38A5 is up-regulated in TNBC cell lines and promotes macropinocytosis, an endocytic process by which cancer cells uptake large amounts of extracellular fluid and its components (Ramachandran et al., 2021). Interestingly, while SLC38A5 enhances macropinocytosis, it significantly reduces cell proliferation. As amino acids enter cancer cells via SLC38A5, H^+^ are exchanged out of the cell, potentially helping to maintain intracellular pH (Ramachandran et al., 2021). Thus, during the initial stages of carcinogenesis, SLC38A5 plays a role in the transport of amino acids to cancer cells, which may be related to the promotion of macropinocytosis. In the early stages of tumorigenesis, SLC38A5 facilitates amino acid transport into cancer cells, possibly linked to its role in promoting macropinocytosis. As the tumor microenvironment becomes acidic, the amino acid transport by SLC38A5 can reverse, leading to the efflux of amino acids and Na^+^ in exchange for extracellular H^+^. In addition, SLC38A5 is highly expressed in breast cancer tissues and is associated with lower patient survival rates. Silencing SLC38A5 inhibits tumor growth in murine breast cancer models and increases BC cell sensitivity to cisplatin (Shen et al., 2024).

Niclosamide, an anti-tapeworm drug, has been shown to inhibit SLC38A5 activity in breast cancer cells by blocking H^+^ efflux, resulting in intracellular acidification (Sennoune et al., 2023). Further studies revealed that pretreatment of TNBC cells with niclosamide reduces the protein expression of SLC38A5 and SLC7A11, induces oxidative stress, lipid peroxidation, and ferroptosis, and significantly suppresses breast cancer growth in mouse xenograft models (Mathew et al., 2024). These findings suggest that targeting SLC38A5, either directly or through agents like niclosamide, could be a promising therapeutic strategy for breast cancer.

### Targeting key amino acid catabolic enzymes

#### GLS inhibitors

Glutaminase is highly expressed in breast cancer and is crucial for tumor growth, with therapeutic efforts targeting glutamine catabolism primarily focusing on GLS. Bis-2-(5-phenylacetamido-1,3,4-thiadiazol-2-yl)ethyl sulfide (BPTES) is the first reported specific and selective inhibitor of GLS1, including KGA and GAC, which induces cell death by inhibiting glutamine uptake in cancer cells (Jeong et al., 2013; Robinson et al., 2007). pretreatment with BPTES in TNBC cell lines can increase sensitivity to chemotherapeutic drugs cisplatin and etoposide, inducing apoptosis (Chen et al., 2016). Compound 968 is another small-molecule specific inhibitor of GLS1, capable of inhibiting carcinogenic transformation in breast cancer cells. When combined with adriamycin (ADR), it can reverse ADR resistance in ADR-resistant MCF-7 cells and synergistically inhibit cell viability (Yang et al., 2021b). However, the latest research indicates that 968 also has moderate selectivity for GLS2 and inhibits the growth of BPTES-resistant breast cancer (Lukey et al., 2019). CB-839 (telaglenastat) is a more potent, selective, and orally bioavailable glutaminase inhibitor. CB-839 inhibits cancer cell proliferation by blocking GLS1 activity in TNBC cells and interfering with glutamine utilization, glutamate synthesis, oxygen consumption, and glutathione levels, as well as intermediates in the tricarboxylic acid cycle (Gross et al., 2014). CB-839 has also shown good antitumor activity and tolerability in a mouse breast cancer xenograft model, enhancing resistance and efficacy when used in combination with paclitaxel (Gross et al., 2014). However, some studies suggest that not all TNBC cell lines respond to CB-839 treatment. CB-839-resistant TNBC cells exhibit increased levels of carnitine palmitoyltransferase 2 (CPT2) protein and CPT1 activity (Reis et al., 2019). The combined use of CB-839 and CPT inhibitors can reduce cell proliferation and migration in CB-839-resistant cells, suggesting that dual GLS-CPT1 inhibition may be a promising therapeutic strategy for TNBC. Currently, CB-839 is undergoing phase I/II clinical trials, with patients showing good tolerability and no significant side effects observed in preclinical trials (Wicker et al., 2021).

#### PHGDH inhibitors

In TNBC cells, upregulated PHGDH expression redirects glucose flux toward serine synthesis. Serine is then converted to glycine to support GSH synthesis, which helps cancer cells detoxify reactive oxygen species (ROS) and resist ROS formation induced by doxorubicin. Inhibiting PHGDH using short hairpin RNA results in doxorubicin-induced oxidative stress and increases the sensitivity of breast cancer cells to doxorubicin. This finding is also validated in mouse tumor models (Zhang and Bai, 2016). Additionally, silencing PHGDH can reverse tamoxifen resistance in estrogen receptor-positive breast cancer cells *in vitro* (Metcalf et al., 2021). Therefore, combining PHGDH inhibitors with chemotherapy drugs may have a synergistic effect, offering a new breast cancer treatment option.

NCT-503 was first reported in 2016 as a noncompetitive, reversible PHGDH inhibitor. NCT-503 was found to bind specifically to the PHGDH-dependent MDA-MB-468 cell line, reducing the growth and weight of xenograft tumors in mice without noticeable toxicity (Pacold et al., 2016). CBR-5884 is another noncompetitive, time-dependent PHGDH inhibitor that disrupts the oligomeric state of PHGDH. CBR-5884 inhibits *de novo* serine synthesis in cancer cells and selectively suppresses the proliferation of breast cancer cell lines with high serine biosynthesis activity (Mullarky et al., 2016). Through screening compounds predicted to bind to the allosteric site of PHGDH and subsequent characterization and validation, the non-NAD^+^ competitive allosteric inhibitor PKUMDL-WQ-2101 was successfully identified to effectively reduce PHGDH enzymatic activity (Wang et al., 2017). PKUMDL-WQ-2101 inhibits *de novo* serine synthesis in PHGDH-amplified breast cancer cell lines and shows anti-tumor activity, reducing the growth of MDA-MB-468 xenograft tumors in mice. However, PHGDH inhibitors have not yet been tested in human clinical trials. As serine is a non-essential amino acid, inhibiting *de novo* serine synthesis within cancer cells might lead to cellular tolerance. Thus, studies propose combining dietary serine and glycine starvation with PHDGH inhibitors, which show synergistic inhibition of one-carbon metabolism and cancer growth in both *in vitro* and *in vivo* experiments (Tajan et al., 2021).

#### SHMT1/2 inhibitors

SHMT is a key mitochondrial enzyme in serine catabolism. SHMT2, in particular, plays an important role as an independent prognostic factor for breast cancer patients (Bernhardt et al., 2017). It converts serine to glycine, providing a one-carbon unit for the one-carbon cycle and participating in nucleotide biosynthesis. Due to the poor pharmacokinetic properties and other limitations of previously developed serine/glycine synthesis inhibitors, they have not yet entered clinical trials, indicating the need for further development of clinically usable drugs targeting this pathway.

Pemetrexed, a clinically applied multi-target antifolate cytotoxic chemotherapeutic drug, has been effective in treating breast cancer and metastatic breast cancer patients previously treated with anthracycline or taxane, with lower toxicity levels (Zhou et al., 2015). Pemetrexed acts on the active site of SHMT and competitively inhibits SHMT (Daidone et al., 2011). Another drug, sertraline, commonly used as an antidepressant, has been found to directly bind SHMT1 and SHMT2, making it a promising competitive dual SHMT1/2 inhibitor with clinical application potential (Geeraerts et al., 2021). Sertraline inhibits SHMT1/2 activity and glycine uptake in breast cancer cells to prevent serine accumulation, thereby demonstrating antitumor activity in a serine/glycine synthesis-addicted xenograft breast cancer mouse model.

#### L-ASNase

The survival and proliferation abilities of breast cancer cells are closely associated with asparagine levels. ASNS catalyzes the production of asparagine from aspartate and glutamine. Due to the lack of ASNS expression in some cancer cells, these cells become dependent on asparagine from serum. L-asparaginase (L-ASNase) has been identified as the first anti-tumor drug directly targeting asparagine metabolism. L-ASNase hydrolyzes L-asparagine into L-ammonia and aspartate, thereby limiting the availability of exogenous L-asparagine (Brumano et al., 2018). Breast cancer cells treated with L-ASNase exhibit significant cytotoxicity and promote a p53-dependent mitochondrial apoptosis pathway (Shahnazari et al., 2022). Currently, L-ASNase is FDA-approved primarily for clinical trials in leukemia and has not yet been further studied in breast cancer patients.

#### ADI-PEG20

Due to the deficiency of ASS1 in breast cancer, the ability of cancer cells to synthesize arginine decreases, causing these cells to become dependent on external arginine or to experience arginine starvation. Arginine deiminase (ADI) is a microbial enzyme isolated from “Mycoplasma,” capable of targeting exogenous arginine and metabolizing it into citrulline, with an effectiveness approximately 300 times that of arginase (Takaku et al., 1992). The antitumor activity of ADI has attracted significant attention in various tumors. Recombinant pegylated ADI (ADI-PEG20), a novel ADI, has undergone multiple Phase I/II clinical trials, demonstrating higher safety and efficacy (Yang et al., 2010). ADI-PEG20 induces extensive mitochondrial dysfunction in various ASS1-deficient breast cancer cell lines, subsequently inducing autophagy. ADI-PEG20 induces arginine starvation in breast cancer xenografts in mice and shows a synergistic effect when used in combination with doxorubicin (Qiu et al., 2014). *In vitro* studies on breast cancer cells have shown that ADI-PEG20 and doxorubicin have synergistic therapeutic effects, with liposomal doxorubicin exhibiting reduced cardiotoxicity. Therefore, a Phase I clinical trial of ADI-PEG20 and liposomal doxorubicin is being conducted in patients with HER2-negative metastatic breast cancer or other advanced solid tumors with ASS1 deficiency (Yao et al., 2022). Although ADI-PEG20 has shown promising efficacy in ASS1-deficient breast cancer cells, resistance to ADI in some ASS1-overexpressing breast cancer cell lines remains a challenge for ADI-based therapy. Consequently, combining ADI-PEG20 with other chemotherapeutic drugs holds promise for effective cancer treatment.

#### IDO1 inhibitors

Extensive research has demonstrated that targeting IDO1 can inhibit the tryptophan metabolism of cancer cells, suggesting IDO1 as a viable drug target for cancer therapy. Indoximod (D-1MT, NLG8189) has garnered widespread attention due to its role in immune evasion by cancer cells and is one of the most studied IDO1 inhibitors in preclinical studies. Its mechanism of action differs from other direct IDO1 enzyme inhibitors and may involve interference with multiple targets within the IDO pathway. In TNBC cells, Indoximod significantly reduces cell viability, and its combination with TNF-α shows superior effects compared to its use alone, with the combination therapy significantly upregulating PD-L1 expression levels (Guney Eskiler and Bilir, 2021). A Phase II clinical trial (NCT01792050) investigated the efficacy of combining Indoximod with the taxane chemotherapy drug Docetaxel in treating metastatic ERBB2-negative breast cancer patients. Unfortunately, the combination therapy did not show a significant difference in efficacy or increased toxicity compared to Docetaxel alone, leading to the premature termination of the study (Mariotti et al., 2021). Another Phase I/II trial (NCT01042535) evaluated the efficacy of Indoximod combined with an adenovirus-p53 transduced dendritic cell vaccine in treating metastatic breast cancer patients (Soliman et al., 2018). A total of 39 patients were included in the study, and 22 patients who received at least two cycles of subsequent vaccine therapy were assessed for response. Of these, nine patients (40%) showed stable or improved disease on imaging. The combination of the two drugs was well-tolerated, and although there was potential for chemopotentiation, the study did not achieve the predefined threshold of a 20% objective response rate.

Epacadostat is an oral, potent, and selective IDO1 inhibitor that prevents immune evasion by enhancing the proliferation of effector T cells and inhibiting their conversion into Tregs, thereby exhibiting strong antitumor activity (Hong et al., 2018). The ECHO202 study is a Phase I/II clinical trial designed to evaluate the efficacy, safety, and tolerability of epacadostat in combination with the PD-1 inhibitor pembrolizumab in patients with advanced or recurrent cancers. In the Phase I trial, of the 62 enrolled patients, only 3 had TNBC. At the end of this phase, only 1 TNBC patient had stable disease (Mitchell et al., 2018). In the Phase II study, among the 382 patients, 36 had TNBC. This cohort included 1 patient with complete response, 1 with very good partial response, 2 with partial response, 7 with stable disease, 19 with disease progression, and 6 whose disease status could not be assessed. TNBC was not considered for inclusion in Phase III studies. Following the failure of the ECHO-301 study, which compared epacadostat plus pembrolizumab to pembrolizumab alone in patients with advanced melanoma and found no significant clinical benefit (Long et al., 2019), several Phase III trials combining epacadostat with immune checkpoint inhibitors were either paused or transitioned to Phase II studies.

Navoximod (GDC-0919) is an oral small-molecule dual inhibitor of IDO1 and TDO. In preclinical tumor models, Navoximod, when combined with anti-PD-L1 therapy, more effectively activates tumor-infiltrating CD8^+^ T cells and inhibits tumor growth in mice compared to single-agent treatments. In a Phase Ia clinical trial (NCT02048709) involving 22 patients with solid tumors, including 1 breast cancer patient, Navoximod was tested. This trial indicated that Navoximod, as a monotherapy, generally had good tolerability but limited antitumor activity (Nayak-Kapoor et al., 2018). Subsequently, in a Phase I study (NCT02471846), the efficacy and safety of Navoximod combined with the PD-L1 inhibitor atezolizumab were evaluated in patients with advanced cancers. Among the 152 recruited patients, 25 had breast cancer. While the combination therapy showed acceptable safety and tolerability, there was no evidence that it provided additional clinical benefit compared to atezolizumab alone (Jung et al., 2019).

### Dietary interventions

Customizing dietary habits based on individual patient profiles offers a promising avenue in amino acid metabolism and breast cancer therapy. Approaches limiting amino acids to tumor tissues through modulating their availability have been tested in preclinical models and clinical trials (Tajan and Vousden, 2020). Specific amino acids in circulation can be selectively limited by either removing them from the diet or utilizing enzymes designed to degrade the targeted amino acid. For example, L-asparaginase encapsulated in erythrocytes to reduce asparagine in plasma is being tested in combination with chemotherapy in patients with locally recurrent or metastatic TNBC (NCT03674242). Arginine-depleting enzyme ADI-PEG20 has been investigated in combination with doxorubicin in HER2-negative metastatic breast cancer in a Phase I trial (NCT01948843). Specialized dietary interventions have also been shown to reduce circulating amino acid levels which may affect therapy response and cancer progression. For example, ingestion of a plant-based diet that contained lower levels of essential and non-essential amino acids reduced serum levels of amino acids in patients with metastatic breast cancer (NCT03045289) (Scales et al., 2024). A fasting-mimicking diet consisting of a 4-day plant-based low amino-acid substitution diet is safe and effective as an adjunct to neoadjuvant chemotherapy in women with HER2-negative stage II/III breast cancer (NCT02126449) (de Groot et al., 2020). In addition, supplementation with specific amino acids, such as BCAAs, cysteine, and theanine, has been shown to improve metabolic profiles and reduce therapy-related side effects in other cancer types (Kikuchi et al., 2016; Tsuchiya et al., 2016). Importantly, further research is needed to confirm that patients with breast cancer could also benefit from this strategy.

Incorporating dietary interventions into treatment plans requires careful stratification of patients based not only on the metabolic vulnerabilities of the tumor but also on the systemic metabolic status of the host. Advanced metabolomics and genomic analyses could identify which patients are likely to benefit from specific dietary modifications. Additionally, combining personalized diets with ongoing therapies, such as chemotherapy or immunotherapy, can create synergistic effects to optimize metabolic and immune-related outcomes. Thus, to effectively modulate diet, it is essential to tailor interventions to the unique features of both the tumor and its treatment.

### Drug delivery

Because certain amino acid transporters are significantly overexpressed on the surface of breast cancer cells, they can be selected as efficient targets for active targeting of tumor-specific nanocarriers, enabling the delivery of modified chemotherapy drugs. Researchers have designed a paclitaxel nanoparticle (SPG25 NPs) by conjugating nanoparticles with glutamate as a ligand for SLC7A5. This nanoparticle has demonstrated good antitumor effects both *in vitro* and *in vivo* in breast cancer cells (Li et al., 2017). In another independent study, catechol-containing SLC7A5 ligands L- and D-dopa were combined with multibranched gold nanoparticles (AuNPs) to mediate selective photothermal ablation of breast cancer cell lines, making the cancer cells more sensitive to the chemotherapy drugs cisplatin and docetaxel (Ong et al., 2017).

Ribociclib (RB) is a CDK4 and CDK6 inhibitor that has been FDA-approved for the treatment of hormone receptor-positive, HER2-negative metastatic breast cancer. Encapsulating RB in liposomes for drug delivery and combining it with the amino acids L-phenylalanine (Phe), L-aspartic acid (Asp), and glutamic acid (Glu) for targeting breast cancer cells that overexpress SLC7A5 has shown that the targeted liposomes inhibit breast cancer cell activity more effectively and shorten the cell cycle compared to free drug (Afsharzadeh et al., 2024). Using specific nanoparticles or liposomes that target SLC7A5 can enhance drug accumulation in cancer tissues, increasing drug bioavailability while reducing accumulation in non-cancer tissues to mitigate toxicity. Further research in preclinical models is necessary to explore these approaches.

### Individualized profiling and patient stratification

Incorporating personalized medicine into amino acid metabolism research is critical for advancing breast cancer treatment strategies. Metabolic, genetic, and molecular phenotyping, combined with patient stratification, enables the development of tailored therapeutic interventions. Recent advancements in multi-layered diagnostic technologies, such as liquid biopsies, metabolic imaging, and AI-driven data analysis, offer opportunities for improved stratification and individualized treatment planning (Acosta et al., 2022; Janssen et al., 2024). High-resolution metabolomics can provide quantitative data on amino acid levels in plasma, tumor tissue, and other biofluids (Danzi et al., 2023). This method can identify metabolic vulnerabilities, such as increased glutamine or serine dependency, guiding therapeutic decisions. Functional imaging modalities, including PET with radiolabeled amino acids (e.g., 18F-FGln PET) and hyperpolarized 13C-MRI, allow for *in vivo* visualization of amino acid metabolism, aiding tumor characterization and monitoring treatment responses (Gallagher et al., 2020). Additionally, analyzing circulating tumor DNA (ctDNA) and circulating tumor cells (CTCs) for mutations in metabolic genes can reveal amino acid dependencies of metastatic or therapy-resistant clones, offering real-time stratification data (Alba-Bernal et al., 2024).

Variations in circulating amino acid levels have been observed between healthy individuals and patients with breast cancer, as well as among breast cancer subtypes, suggesting a potential for individualizing profiling (Fan et al., 2016; Shen et al., 2013). In this regard, AI integration considering multi-omics parameters including metabolomics enhances predictive strategies, while digital health monitoring can track real-time metabolic changes (Miller et al., 2019). These innovations may significantly improve prognoses by enabling personalized interventions, facilitating a shift toward therapies targeting specific metabolic and immune vulnerabilities in breast cancer subtypes.

The distinct characteristics of amino acid metabolism offer a framework for predictive, preventive, and personalized medicine (3PM), providing insights into metabolic vulnerabilities and therapeutic opportunities (Pesta et al., 2024). Stratified amino acid profiling and metabolic phenotyping aid in identifying specific metabolic reprogramming, supporting patient stratification, and enabling three distinct levels of prevention: primary, secondary, and tertiary.

#### Primary prevention—Early intervention for high-risk populations

For individuals with modifiable risk factors, such as obesity, chronic inflammation, or preclinical metabolic disorders, amino acid analysis can detect early metabolic shifts indicative of breast cancer susceptibility (Brown, 2021). Elevated levels of key components in biosynthetic and redox pathways, such as glutamine or serine, may signal an increased risk of breast cancer. Dietary and lifestyle interventions targeting amino acid pathways, such as serine/glycine restriction or protein intake modulation, could mitigate these risks (Zhang et al., 2025). Exercise and weight control can further reduce amino acid-driven anabolic processes, particularly in populations with metabolic syndrome or chronic inflammation.

#### Secondary prevention—Targeting early disease

For patients with non-modifiable risk factors, such as genetic predispositions (e.g., BRCA mutations) or mitochondrial dysfunction, metabolic phenotyping can identify actionable targets. Tumors with BRCA mutations often depend on glutamine metabolism for growth and survival. Analyzing pathways involving amino acid transporters (e.g., SLC1A5) or enzymes (e.g., GLS) can guide the use of glutaminase inhibitors, such as CB-839, for glutamine-addicted breast cancer (Wicker et al., 2021). Similarly, mitochondrial dysfunction linked to altered aspartate metabolism can be treated through interventions targeting compensatory pathways (Cheng et al., 2018). Thus, early tumor metabolic phenotyping through CTCs enables stratification based on metabolic vulnerabilities, promoting personalized prevention and treatment strategies.

#### Tertiary prevention—Personalized therapies for aggressive subtypes

In metastatic or therapy-resistant breast cancer, amino acid reprogramming is crucial for sustaining tumor growth and adapting to therapeutic pressures. For instance, TNBC often exhibits dependency on BCAA metabolism or upregulation of the serine synthesis pathway (Huang et al., 2024; Luo et al., 2024). Amino acid analysis in advanced tumors can guide the application of novel therapies, such as PHGDH inhibitors for serine-dependent cancers or targeting asparagine metabolism to reduce metastatic spread (Knott et al., 2018). Furthermore, tryptophan metabolism through the kynurenine pathway suppresses anti-tumor immunity, and targeting IDO1 can enhance the efficacy of immune checkpoint inhibitors in advanced cancers (Triplett et al., 2018). Combining amino acid-targeted drugs with chemotherapy, radiotherapy, or dietary restrictions can overcome resistance by disrupting cancer cell survival mechanisms, such as redox balance or anabolic growth.

By addressing all levels of prevention, these strategies create a comprehensive framework for improving breast cancer outcomes and advancing precision medicine.

## Conclusion and expert recommendations

Amino acid metabolism plays a pivotal role in breast cancer biology, significantly influencing tumor growth, metastasis, and therapy resistance. Dysregulation of amino acid transporters and subsequent metabolic reprogramming within cancer cells highlights promising therapeutic targets. However, a critical challenge lies in the potential compensatory mechanisms that cancer cells may employ, such as upregulating other amino acid transporters to overcome the inhibition of a specific transporter, which may limit the long-term efficacy of single-agent therapies. To address these challenges, future research should prioritize combination treatment approaches that simultaneously target multiple amino acid transporters or metabolic pathways. For instance, combining metabolic inhibitors with immunotherapy, chemotherapy, or radiotherapy could yield synergistic effects by leveraging the vulnerabilities of cancer cells while minimizing compensatory adaptations.

Another essential direction for research involves unraveling the interplay between amino acid metabolism and the tumor microenvironment, including stromal cells and immune infiltrates. A deeper understanding of this interaction is critical for designing therapies that address both metabolic and immune-related weaknesses. Furthermore, patient stratification and personalized medicine represent a critical frontier. Advanced tools, such as metabolomics, functional imaging, and genomic profiling, could enable precise classification of patients based on their unique metabolic phenotypes. It could guide individualized therapeutic strategies.

AI offers transformative potential in this field by integrating complex datasets, including genomics, transcriptomics, metabolomics, and imaging data, to optimize patient stratification and treatment plans. AI-driven systems can identify metabolic biomarkers, predict therapeutic responses, and model potential compensatory mechanisms in cancer cells. Additionally, AI-based monitoring tools could track dynamic metabolic changes during treatment, allowing for real-time adjustments to maximize therapeutic efficacy.

To translate these insights into clinical practice, several recommendations emerge. Amino acid analysis and metabolic phenotyping could be developed as a strategy for diagnostics to identify metabolic vulnerabilities in breast cancer patients earlier. AI-assisted predictive models should be utilized to customize metabolic therapies based on individual tumor characteristics, taking into account the heterogeneity of breast cancer subtypes. Clinical trials should prioritize combining metabolic inhibitors with immunotherapies, chemotherapies, and radiotherapies to evaluate their efficacy in overcoming resistance. Importantly, therapeutic interventions modulating amino acid levels can directly impact mitochondrial function by reducing the availability of substrates critical for the TCA cycle. Thus, a personalized rehabilitation strategy addressing mitochondrial health could be particularly relevant for breast cancer patients who undergo amino acid metabolism-targeted therapies (Golubnitschaja et al., 2024; Pesta et al., 2024). Holistic rehabilitation programs incorporating dietary interventions, physical activity, and mitochondrial support nutraceuticals need to be further assessed as approaches to improve metabolic health and overall well-being in these cases.

Personalized medicine in amino acid metabolism research is essential for addressing the unique challenges of breast cancer subtypes. Generalized treatments fail to capture the heterogeneity of these cancers, underscoring the need for patient stratification based on metabolic phenotypes. Vulnerabilities, such as glutamine or serine dependence, represent potential targets but are complicated by cancer cells’ ability to adapt via compensatory pathways. Advances in high-resolution metabolomics, functional imaging, and liquid biopsy offer opportunities to detect these weaknesses, while AI-driven integration of multi-omics data supports the design of tailored therapies. By combining these approaches, we can develop more effective, individualized treatments that improve patient outcomes and quality of life. Moving forward, integrating personalized medicine with amino acid metabolism research provides a roadmap to overcome current limitations and achieve precision oncology for breast cancer patients.

## References

[CIT0001] Acosta JN , Falcone GJ, Rajpurkar P et al Multimodal biomedical AI. Nat Med 2022;28:1773–1784. 36109635 10.1038/s41591-022-01981-2

[CIT0002] Afsharzadeh M , Varshosaz J, Mirian M et al Targeted delivery of liposomal Ribociclib to SLC7A5 transporters in breast cancer cells. Invest New Drugs 2024;42:89–105. 38127209 10.1007/s10637-023-01409-9

[CIT0003] Alba-Bernal A , Godoy-Ortiz A, Dominguez-Recio ME et al Increased blood draws for ultrasensitive ctDNA and CTCs detection in early breast cancer patients. NPJ Breast Cancer 2024;10:36. 38750090 10.1038/s41523-024-00642-6 PMC11096188

[CIT0004] Alcin G , Tatar G, Menengic Koc MS et al Complex fibroadenoma mimicking breast cancer on ^68^Ga-FAPI-04 and ^18^F-FDG PET/CT. Clin Nucl Med 2023;48:e121–e123. 36723896 10.1097/RLU.0000000000004495

[CIT0005] Alfarsi LH , El Ansari R, Craze ML et al SLC1A5 co-expression with TALDO1 associates with endocrine therapy failure in estrogen receptor-positive breast cancer. Breast Cancer Res Treat 2021;189:317–331. 34282517 10.1007/s10549-021-06298-1 PMC8357718

[CIT0006] Altman BJ , Stine ZE, Dang CV. From Krebs to clinic: glutamine metabolism to cancer therapy. Nat Rev Cancer 2016;16:619–634. 27492215 10.1038/nrc.2016.71 PMC5484415

[CIT0007] Babu E , Bhutia YD, Ramachandran S et al Deletion of the amino acid transporter Slc6a14 suppresses tumour growth in spontaneous mouse models of breast cancer. Biochem J 2015;469:17–23. 26173258 10.1042/BJ20150437

[CIT0008] Bacci M , Lorito N, Ippolito L et al Reprogramming of amino acid transporters to support aspartate and glutamate dependency sustains endocrine resistance in breast cancer. Cell Rep 2019;28:104–118.e8. 31269432 10.1016/j.celrep.2019.06.010 PMC6616584

[CIT0009] Baek S , Choi CM, Ahn SH et al Exploratory clinical trial of (4S)-4-(3-[18F]fluoropropyl)-L-glutamate for imaging xC- transporter using positron emission tomography in patients with non-small cell lung or breast cancer. Clin Cancer Res 2012;18:5427–5437. 22893629 10.1158/1078-0432.CCR-12-0214

[CIT0010] Barnabas GD , Lee JS, Shami T et al Serine biosynthesis is a metabolic vulnerability in IDH2-driven breast cancer progression. Cancer Res 2021;81:1443–1456. 33500247 10.1158/0008-5472.CAN-19-3020 PMC9216326

[CIT0011] Bernhardt S , Bayerlova M, Vetter M et al Proteomic profiling of breast cancer metabolism identifies SHMT2 and ASCT2 as prognostic factors. Breast Cancer Res 2017;19:112. 29020998 10.1186/s13058-017-0905-7 PMC5637318

[CIT0012] Bray F , Laversanne M, Sung H et al Global cancer statistics 2022: GLOBOCAN estimates of incidence and mortality worldwide for 36 cancers in 185 countries. CA Cancer J Clin 2024;74:229–263. 38572751 10.3322/caac.21834

[CIT0013] Broer A , Fairweather S, Broer S. Disruption of amino acid homeostasis by novel ASCT2 inhibitors involves multiple targets. Front Pharmacol 2018;9:785. 30072900 10.3389/fphar.2018.00785 PMC6060247

[CIT0014] Brown KA. Metabolic pathways in obesity-related breast cancer. Nat Rev Endocrinol 2021;17:350–363. 33927368 10.1038/s41574-021-00487-0 PMC10410950

[CIT0015] Brumano LP , da Silva FVS, Costa-Silva TA et al Development of L-Asparaginase Biobetters: current research status and review of the desirable quality profiles. Front Bioeng Biotechnol 2018;6:212. 30687702 10.3389/fbioe.2018.00212 PMC6335324

[CIT0018] Chen Z , Wang Y, Warden C et al Cross-talk between ER and HER2 regulates c-MYC-mediated glutamine metabolism in aromatase inhibitor resistant breast cancer cells. J Steroid Biochem Mol Biol 2015;149:118–127. 25683269 10.1016/j.jsbmb.2015.02.004 PMC4380584

[CIT0017] Chen L , Cui H, Fang J et al Glutamine deprivation plus BPTES alters etoposide- and cisplatin-induced apoptosis in triple negative breast cancer cells. Oncotarget 2016;7:54691–54701. 27419628 10.18632/oncotarget.10579 PMC5342373

[CIT0016] Chen CL , Hsu SC, Ann DK et al Arginine signaling and cancer metabolism. Cancers (Basel) 2021;13:3541. 34298755 10.3390/cancers13143541 PMC8306961

[CIT0019] Cheng CT , Qi Y, Wang YC et al Arginine starvation kills tumor cells through aspartate exhaustion and mitochondrial dysfunction. Commun Biol 2018;1:178. 30393775 10.1038/s42003-018-0178-4 PMC6203837

[CIT0020] Chi R , Yao C, Chen S et al Elevated BCAA suppresses the development and metastasis of breast cancer. Front Oncol 2022;12:887257. 35785192 10.3389/fonc.2022.887257 PMC9243538

[CIT0021] Cluntun AA , Lukey MJ, Cerione RA et al Glutamine metabolism in cancer: understanding the heterogeneity. Trends Cancer 2017;3:169–180. 28393116 10.1016/j.trecan.2017.01.005 PMC5383348

[CIT0022] Coloff JL , Murphy JP, Braun CR et al Differential glutamate metabolism in proliferating and quiescent mammary epithelial cells. Cell Metab 2016;23:867–880. 27133130 10.1016/j.cmet.2016.03.016

[CIT0023] Colombero C , Remy D, Antoine-Bally S et al mTOR repression in response to amino acid starvation promotes ECM degradation through MT1-MMP endocytosis arrest. Adv Sci (Weinh) 2021;8:e2101614. 34250755 10.1002/advs.202101614 PMC8425857

[CIT0024] Conrad M , Sato H. The oxidative stress-inducible cystine/glutamate antiporter, system x (^c^)(^-^): cystine supplier and beyond. Amino Acids 2012;42:231–246. 21409388 10.1007/s00726-011-0867-5

[CIT0025] Cormerais Y , Massard PA, Vucetic M et al The glutamine transporter ASCT2 (SLC1A5) promotes tumor growth independently of the amino acid transporter LAT1 (SLC7A5). J Biol Chem 2018;293:2877–2887. 29326164 10.1074/jbc.RA117.001342 PMC5827425

[CIT0026] D’Amato NC , Rogers TJ, Gordon MA et al A TDO2-AhR signaling axis facilitates anoikis resistance and metastasis in triple-negative breast cancer. Cancer Res 2015;75:4651–4664. 26363006 10.1158/0008-5472.CAN-15-2011 PMC4631670

[CIT0027] Daidone F , Florio R, Rinaldo S et al In silico and in vitro validation of serine hydroxymethyltransferase as a chemotherapeutic target of the antifolate drug pemetrexed. Eur J Med Chem 2011;46:1616–1621. 21371789 10.1016/j.ejmech.2011.02.009

[CIT0028] Danzi F , Pacchiana R, Mafficini A et al To metabolomics and beyond: a technological portfolio to investigate cancer metabolism. Signal Transduct Target Ther 2023;8:137. 36949046 10.1038/s41392-023-01380-0 PMC10033890

[CIT0029] de Groot S , Lugtenberg RT, Cohen D et al Fasting mimicking diet as an adjunct to neoadjuvant chemotherapy for breast cancer in the multicentre randomized phase 2 DIRECT trial. Nat Commun 2020;11:3083. 32576828 10.1038/s41467-020-16138-3 PMC7311547

[CIT0030] Deng L , Yao P, Li L et al p53-mediated control of aspartate-asparagine homeostasis dictates LKB1 activity and modulates cell survival. Nat Commun 2020;11:1755. 32273511 10.1038/s41467-020-15573-6 PMC7145870

[CIT0031] Dias MM , Adamoski D, Dos Reis LM et al GLS2 is protumorigenic in breast cancers. Oncogene 2020;39:690–702. 31541193 10.1038/s41388-019-1007-z

[CIT0032] Doglioni G , Fernandez-Garcia J, Igelmann S et al Aspartate signalling drives lung metastasis via alternative translation. Nature 2025;638:244–250. 39743589 10.1038/s41586-024-08335-7 PMC7618879

[CIT0033] Dong J , Xiao D, Zhao Z et al Epigenetic silencing of microRNA-137 enhances ASCT2 expression and tumor glutamine metabolism. Oncogenesis 2017;6:e356. 28692032 10.1038/oncsis.2017.59 PMC5541711

[CIT0035] El Ansari R , Craze ML, Miligy I et al The amino acid transporter SLC7A5 confers a poor prognosis in the highly proliferative breast cancer subtypes and is a key therapeutic target in luminal B tumours. Breast Cancer Res 2018;20:21. 29566741 10.1186/s13058-018-0946-6 PMC5863851

[CIT0034] El-Botty R , Morriset L, Montaudon E et al Oxidative phosphorylation is a metabolic vulnerability of endocrine therapy and palbociclib resistant metastatic breast cancers. Nat Commun 2023;14:4221. 37452026 10.1038/s41467-023-40022-5 PMC10349040

[CIT0036] Fan Y , Zhou X, Xia TS et al Human plasma metabolomics for identifying differential metabolites and predicting molecular subtypes of breast cancer. Oncotarget 2016;7:9925–9938. 26848530 10.18632/oncotarget.7155 PMC4891093

[CIT0037] Feng S , Aplin C, Nguyen TT et al Filament formation drives catalysis by glutaminase enzymes important in cancer progression. Nat Commun 2024;15:1971. 38438397 10.1038/s41467-024-46351-3 PMC10912226

[CIT0038] Gallagher FA , Woitek R, McLean MA et al Imaging breast cancer using hyperpolarized carbon-13 MRI. Proc Natl Acad Sci U S A 2020;117:2092–2098. 31964840 10.1073/pnas.1913841117 PMC6995024

[CIT0039] Gao P , Tchernyshyov I, Chang TC et al c-Myc suppression of miR-23a/b enhances mitochondrial glutaminase expression and glutamine metabolism. Nature 2009;458:762–765. 19219026 10.1038/nature07823 PMC2729443

[CIT0040] Garcia-Bermudez J , Baudrier L, La K et al Aspartate is a limiting metabolite for cancer cell proliferation under hypoxia and in tumours. Nat Cell Biol 2018;20:775–781. 29941933 10.1038/s41556-018-0118-z PMC6030478

[CIT0041] Gauthier-Coles G , Broer A, McLeod MD et al Identification and characterization of a novel SNAT2 (SLC38A2) inhibitor reveals synergy with glucose transport inhibition in cancer cells. Front Pharmacol 2022;13:963066. 36210829 10.3389/fphar.2022.963066 PMC9532951

[CIT0042] Geeraerts SL , Kampen KR, Rinaldi G et al Repurposing the antidepressant sertraline as SHMT inhibitor to suppress serine/glycine synthesis-addicted breast tumor growth. Mol Cancer Ther 2021;20:50–63. 33203732 10.1158/1535-7163.MCT-20-0480 PMC7611204

[CIT0043] Golubnitschaja O , Kapinova A, Sargheini N et al Mini-encyclopedia of mitochondria-relevant nutraceuticals protecting health in primary and secondary care-clinically relevant 3PM innovation. EPMA J 2024;15:163–205. 38841620 10.1007/s13167-024-00358-4 PMC11148002

[CIT0044] Gorgoglione R , Impedovo V, Riley CL et al Glutamine-derived aspartate biosynthesis in cancer cells: role of mitochondrial transporters and new therapeutic perspectives. Cancers (Basel) 2022;14:245. 35008407 10.3390/cancers14010245 PMC8750728

[CIT0045] Gross MI , Demo SD, Dennison JB et al Antitumor activity of the glutaminase inhibitor CB-839 in triple-negative breast cancer. Mol Cancer Ther 2014;13:890–901. 24523301 10.1158/1535-7163.MCT-13-0870

[CIT0046] Guney Eskiler G , Bilir C. The efficacy of indoximod upon stimulation with pro-inflammatory cytokines in triple-negative breast cancer cells. Immunopharmacol Immunotoxicol 2021;43:554–561. 34314307 10.1080/08923973.2021.1953064

[CIT0047] Hickok JR , Vasudevan D, Antholine WE et al Nitric oxide modifies global histone methylation by inhibiting Jumonji C domain-containing demethylases. J Biol Chem 2013;288:16004–16015. 23546878 10.1074/jbc.M112.432294 PMC3668755

[CIT0049] Hong R , Zhou Y, Tian X et al Selective inhibition of IDO1, D-1-methyl-tryptophan (D-1MT), effectively increased EpCAM/CD3-bispecific BiTE antibody MT110 efficacy against IDO1(hi)breast cancer via enhancing immune cells activity. Int Immunopharmacol 2018;54:118–124. 29128855 10.1016/j.intimp.2017.10.008

[CIT0048] Hong R , Zhang W, Xia X et al Preventing BRCA1/ZBRK1 repressor complex binding to the GOT2 promoter results in accelerated aspartate biosynthesis and promotion of cell proliferation. Mol Oncol 2019;13:959–977. 30714292 10.1002/1878-0261.12466 PMC6441895

[CIT0050] Hsu WJ , Chiang MC, Chao YC et al Arginine methylation of DDX3 by PRMT1 mediates mitochondrial homeostasis to promote breast cancer metastasis. Cancer Res 2024;84:3023–3043. 39042374 10.1158/0008-5472.CAN-23-3829

[CIT0052] Huang R , Wang H, Hong J et al Targeting glutamine metabolic reprogramming of SLC7A5 enhances the efficacy of anti-PD-1 in triple-negative breast cancer. Front Immunol 2023;14:1251643. 37731509 10.3389/fimmu.2023.1251643 PMC10507177

[CIT0051] Huang L , Li G, Zhang Y et al Small-molecule targeting BCAT1-mediated BCAA metabolism inhibits the activation of SHOC2-RAS-ERK to induce apoptosis of Triple-negative breast cancer cells. J Adv Res 2024;24:476. 10.1016/j.jare.2024.10.021 PMC12789772 39490614

[CIT0053] Issaq SH , Mendoza A, Fox SD et al Glutamine synthetase is necessary for sarcoma adaptation to glutamine deprivation and tumor growth. Oncogenesis 2019;8:20. 30808861 10.1038/s41389-019-0129-z PMC6391386

[CIT0054] Jacquemier J , Bertucci F, Finetti P et al High expression of indoleamine 2,3-dioxygenase in the tumour is associated with medullary features and favourable outcome in basal-like breast carcinoma. Int J Cancer 2012;130:96–104. 21328335 10.1002/ijc.25979

[CIT0055] Janssen FW , Lak NSM, Janda CY et al A comprehensive overview of liquid biopsy applications in pediatric solid tumors. npj Precis Oncol 2024;8:172. 39097671 10.1038/s41698-024-00657-z PMC11297996

[CIT0056] Jeon YJ , Khelifa S, Ratnikov B et al Regulation of glutamine carrier proteins by RNF5 determines breast cancer response to ER stress-inducing chemotherapies. Cancer Cell 2015;27:354–369. 25759021 10.1016/j.ccell.2015.02.006 PMC4356903

[CIT0057] Jeong SM , Xiao C, Finley LW et al SIRT4 has tumor-suppressive activity and regulates the cellular metabolic response to DNA damage by inhibiting mitochondrial glutamine metabolism. Cancer Cell 2013;23:450–463. 23562301 10.1016/j.ccr.2013.02.024 PMC3650305

[CIT0058] Jhaveri K , Modi S. Ganetespib: research and clinical development. Onco Targets Ther 2015;8:1849–1858. 26244021 10.2147/OTT.S65804 PMC4521669

[CIT0059] Jiao Z , Pan Y, Chen F. The metabolic landscape of breast cancer and its therapeutic implications. Mol Diagn Ther 2023;27:349–369. 36991275 10.1007/s40291-023-00645-2

[CIT0060] Jin J , Byun JK, Choi YK et al Targeting glutamine metabolism as a therapeutic strategy for cancer. Exp Mol Med 2023;55:706–715. 37009798 10.1038/s12276-023-00971-9 PMC10167356

[CIT0061] Jung KH , LoRusso P, Burris H et al Phase I study of the Indoleamine 2,3-Dioxygenase 1 (IDO1) Inhibitor Navoximod (GDC-0919) administered with PD-L1 Inhibitor (Atezolizumab) in advanced solid tumors. Clin Cancer Res 2019;25:3220–3228. 30770348 10.1158/1078-0432.CCR-18-2740 PMC7980952

[CIT0062] Jung MK , Okekunle AP, Lee JE et al Role of branched-chain amino acid metabolism in tumor development and progression. J Cancer Prev 2021;26:237–243. 35047449 10.15430/JCP.2021.26.4.237 PMC8749315

[CIT0063] Karunakaran S , Ramachandran S, Coothankandaswamy V et al SLC6A14 (ATB0,+) protein, a highly concentrative and broad specific amino acid transporter, is a novel and effective drug target for treatment of estrogen receptor-positive breast cancer. J Biol Chem 2011;286:31830–31838. 21771784 10.1074/jbc.M111.229518 PMC3173074

[CIT0064] Keshet R , Erez A. Arginine and the metabolic regulation of nitric oxide synthesis in cancer. Dis Model Mech 2018;11:dmm033332. 30082427 10.1242/dmm.033332 PMC6124554

[CIT0065] Kikuchi Y , Hiroshima Y, Matsuo K et al A randomized clinical trial of preoperative administration of branched-chain amino acids to prevent postoperative ascites in patients with liver resection for hepatocellular carcinoma. Ann Surg Oncol 2016;23:3727–3735. 27338747 10.1245/s10434-016-5348-3

[CIT0066] Kim GW , Lee DH, Jeon YH et al Glutamine synthetase as a therapeutic target for cancer treatment. Int J Mol Sci 2021;22:1701. 33567690 10.3390/ijms22041701 PMC7915753

[CIT0067] Knott SRV , Wagenblast E, Khan S et al Asparagine bioavailability governs metastasis in a model of breast cancer. Nature 2018;554:378–381. 29414946 10.1038/nature25465 PMC5898613

[CIT0068] Koppula P , Zhang Y, Zhuang L et al Amino acid transporter SLC7A11/xCT at the crossroads of regulating redox homeostasis and nutrient dependency of cancer. Cancer Commun (Lond) 2018;38:12. 29764521 10.1186/s40880-018-0288-x PMC5993148

[CIT0069] Kottakis F , Nicolay BN, Roumane A et al LKB1 loss links serine metabolism to DNA methylation and tumorigenesis. Nature 2016;539:390–395. 27799657 10.1038/nature20132 PMC5988435

[CIT0070] Lee G , Wong C, Cho A et al E-cadherin induces serine synthesis to support progression and metastasis of breast cancer. Cancer Res 2024;84:2820–2835. 38959339 10.1158/0008-5472.CAN-23-3082 PMC11374473

[CIT0071] Lei M , Zhang YL, Huang FY et al Gankyrin inhibits ferroptosis through the p53/SLC7A11/GPX4 axis in triple-negative breast cancer cells. Sci Rep 2023;13:21916. 38081931 10.1038/s41598-023-49136-8 PMC10713534

[CIT0072] Levina V , Su Y, Gorelik E. Immunological and nonimmunological effects of indoleamine 2,3-dioxygenase on breast tumor growth and spontaneous metastasis formation. Clin Dev Immunol 2012;2012:173029. 22654951 10.1155/2012/173029 PMC3359678

[CIT0073] Li L , Di X, Wu M et al Targeting tumor highly-expressed LAT1 transporter with amino acid-modified nanoparticles: Toward a novel active targeting strategy in breast cancer therapy. Nanomedicine 2017;13:987–998. 27890657 10.1016/j.nano.2016.11.012

[CIT0074] Li Y , Wang W, Wu X et al SLC7A5 serves as a prognostic factor of breast cancer and promotes cell proliferation through activating AKT/mTORC1 signaling pathway. Ann Transl Med 2021;9:892. 34164526 10.21037/atm-21-2247 PMC8184433

[CIT0075] Liang Z , Cho HT, Williams L et al Potential biomarker of L-type amino acid transporter 1 in breast cancer progression. Nucl Med Mol Imaging 2011;45:93–102. 24899987 10.1007/s13139-010-0068-2 PMC4043027

[CIT0076] Lieu EL , Nguyen T, Rhyne S et al Amino acids in cancer. Exp Mol Med 2020;52:15–30. 31980738 10.1038/s12276-020-0375-3 PMC7000687

[CIT0077] Ling ZN , Jiang YF, Ru JN et al Amino acid metabolism in health and disease. Signal Transduct Target Ther 2023;8:345. 37699892 10.1038/s41392-023-01569-3 PMC10497558

[CIT0078] Liu X , Ren B, Ren J et al The significant role of amino acid metabolic reprogramming in cancer. Cell Commun Signal2024a;22:380. 39069612 10.1186/s12964-024-01760-1 PMC11285422

[CIT0079] Liu Y , Chen S, Wan X et al Tryptophan 2,3-dioxygenase-positive matrix fibroblasts fuel breast cancer lung metastasis via kynurenine-mediated ferroptosis resistance of metastatic cells and T cell dysfunction. Cancer Commun (Lond) 2024b;44:1261–1286. 39221971 10.1002/cac2.12608 PMC11570772

[CIT0080] Locasale JW , Cantley LC. Genetic selection for enhanced serine metabolism in cancer development. Cell Cycle 2011;10:3812–3813. 22064516 10.4161/cc.10.22.18224

[CIT0081] Loibl S , André F, Bachelot T et al Early breast cancer: ESMO Clinical Practice Guideline for diagnosis, treatment and follow-up. Ann Oncol 2024;35:159–182. 38101773 10.1016/j.annonc.2023.11.016

[CIT0082] Long GV , Dummer R, Hamid O et al Epacadostat plus pembrolizumab versus placebo plus pembrolizumab in patients with unresectable or metastatic melanoma (ECHO-301/KEYNOTE-252): a phase 3, randomised, double-blind study. Lancet Oncol 2019;20:1083–1097. 31221619 10.1016/S1470-2045(19)30274-8

[CIT0083] Lukey MJ , Cluntun AA, Katt WP et al Liver-Type Glutaminase GLS2 Is a druggable metabolic node in luminal-subtype breast cancer. Cell Rep 2019;29:76–88.e7. 31577957 10.1016/j.celrep.2019.08.076 PMC6939472

[CIT0084] Luo W , Zou Z, Nie Y et al ASS1 inhibits triple-negative breast cancer by regulating PHGDH stability and de novo serine synthesis. Cell Death Dis 2024;15:319. 38710705 10.1038/s41419-024-06672-z PMC11074131

[CIT0085] Mariotti V , Han H, Ismail-Khan R et al Effect of taxane chemotherapy with or without indoximod in metastatic breast cancer: a randomized clinical trial. JAMA Oncol 2021;7:61–69. 33151286 10.1001/jamaoncol.2020.5572 PMC7645745

[CIT0086] Maris M , Salles G, Kim WS et al ASCT2-targeting antibody-drug conjugate MEDI7247 in adult patients with relapsed/refractory hematological malignancies: a first-in-human, Phase 1 study. Target Oncol 2024;19:321–332. 38683495 10.1007/s11523-024-01054-z PMC11111564

[CIT0087] Masisi BK , El Ansari R, Alfarsi L et al The biological and clinical significance of glutaminase in luminal breast cancer. Cancers (Basel) 2021;13:3963. 34439127 10.3390/cancers13163963 PMC8391318

[CIT0088] Mathew M , Sivaprakasam S, Dharmalingam-Nandagopal G et al Induction of oxidative stress and ferroptosis in triple-negative breast cancer cells by niclosamide via blockade of the function and expression of SLC38A5 and SLC7A11. Antioxidants (Basel) 2024;13:291. 38539825 10.3390/antiox13030291 PMC10967572

[CIT0089] Meireson A , Devos M, Brochez L. IDO expression in cancer: different compartment, different functionality? Front Immunol 2020;11:531491. 33072086 10.3389/fimmu.2020.531491 PMC7541907

[CIT0090] Metcalf S , Petri BJ, Kruer T et al Serine synthesis influences tamoxifen response in ER+ human breast carcinoma. Endocr Relat Cancer 2021;28:27–37. 33112838 10.1530/ERC-19-0510 PMC7780089

[CIT0091] Miller IJ , Peters SR, Overmyer KA et al Real-time health monitoring through urine metabolomics. NPJ Digit Med 2019;2:109. 31728416 10.1038/s41746-019-0185-y PMC6848197

[CIT0092] Mitchell TC , Hamid O, Smith DC et al Epacadostat Plus Pembrolizumab in patients with advanced solid tumors: phase I results from a multicenter, open-label Phase I/II Trial (ECHO-202/KEYNOTE-037). J Clin Oncol 2018;36:3223–3230. 30265610 10.1200/JCO.2018.78.9602 PMC6225502

[CIT0093] Morotti M , Bridges E, Valli A et al Hypoxia-induced switch in SNAT2/SLC38A2 regulation generates endocrine resistance in breast cancer. Proc Natl Acad Sci U S A 2019;116:12452–12461. 31152137 10.1073/pnas.1818521116 PMC6589752

[CIT0094] Morotti M , Zois CE, El-Ansari R et al Increased expression of glutamine transporter SNAT2/SLC38A2 promotes glutamine dependence and oxidative stress resistance, and is associated with worse prognosis in triple-negative breast cancer. Br J Cancer 2021;124:494–505. 33028955 10.1038/s41416-020-01113-y PMC7852531

[CIT0095] Mullarky E , Lucki NC, Beheshti Zavareh R et al Identification of a small molecule inhibitor of 3-phosphoglycerate dehydrogenase to target serine biosynthesis in cancers. Proc Natl Acad Sci U S A 2016;113:1778–1783. 26831078 10.1073/pnas.1521548113 PMC4763784

[CIT0096] Nalecz KA. Amino Acid Transporter SLC6A14 (ATB^0,+^) – A target in combined anti-cancer therapy. Front Cell Dev Biol 2020;8:594464. 33195271 10.3389/fcell.2020.594464 PMC7609839

[CIT0097] Nath P , Alfarsi LH, El-Ansari R et al The amino acid transporter SLC7A11 expression in breast cancer. Cancer Biol Ther 2024;25:2291855. 38073087 10.1080/15384047.2023.2291855 PMC10761065

[CIT0098] Nayak-Kapoor A , Hao Z, Sadek R et al Phase Ia study of the indoleamine 2,3-dioxygenase 1 (IDO1) inhibitor navoximod (GDC-0919) in patients with recurrent advanced solid tumors. J ImmunoTher Cancer 2018;6:61. 29921320 10.1186/s40425-018-0351-9 PMC6009946

[CIT0099] Novikov O , Wang Z, Stanford EA et al An aryl hydrocarbon receptor-mediated amplification loop that enforces cell migration in ER^–^PR^–^/Her2^-^ human breast cancer cells. Mol Pharmacol 2016;90:674–688. 27573671 10.1124/mol.116.105361 PMC5074452

[CIT0100] Oda K , Hosoda N, Endo H et al L-type amino acid transporter 1 inhibitors inhibit tumor cell growth. Cancer Sci 2010;101:173–179. 19900191 10.1111/j.1349-7006.2009.01386.x PMC11158286

[CIT0101] Onesti CE , Boemer F, Josse C et al Tryptophan catabolism increases in breast cancer patients compared to healthy controls without affecting the cancer outcome or response to chemotherapy. J Transl Med 2019;17:239. 31337401 10.1186/s12967-019-1984-2 PMC6652004

[CIT0102] Ong ZY , Chen S, Nabavi E et al Multibranched gold nanoparticles with intrinsic LAT-1 targeting capabilities for selective photothermal therapy of breast cancer. ACS Appl Mater Interfaces 2017;9:39259–39270. 29058874 10.1021/acsami.7b14851

[CIT0103] Pacold ME , Brimacombe KR, Chan SH et al A PHGDH inhibitor reveals coordination of serine synthesis and one-carbon unit fate. Nat Chem Biol 2016;12:452–458. 27110680 10.1038/nchembio.2070 PMC4871733

[CIT0104] Parida PK , Marquez-Palencia M, Nair V et al Metabolic diversity within breast cancer brain-tropic cells determines metastatic fitness. Cell Metab 2022;34:90–105.e7. 34986341 10.1016/j.cmet.2021.12.001 PMC9307073

[CIT0105] Pesta M , Mrazova B, Kapalla M et al Mitochondria-based holistic 3PM approach as the ‘game-changer’ for individualised rehabilitation-the proof-of-principle model by treated breast cancer survivors. EPMA J 2024;15:559–571. 39635015 10.1007/s13167-024-00386-0 PMC11612048

[CIT0106] Platten M , Nollen EAA, Rohrig UF et al Tryptophan metabolism as a common therapeutic target in cancer, neurodegeneration and beyond. Nat Rev Drug Discov 2019;18:379–401. 30760888 10.1038/s41573-019-0016-5

[CIT0107] Pollari S , Kakonen SM, Edgren H et al Enhanced serine production by bone metastatic breast cancer cells stimulates osteoclastogenesis. Breast Cancer Res Treat 2011;125:421–430. 20352489 10.1007/s10549-010-0848-5

[CIT0108] Possemato R , Marks KM, Shaul YD et al Functional genomics reveal that the serine synthesis pathway is essential in breast cancer. Nature 2011;476:346–350. 21760589 10.1038/nature10350 PMC3353325

[CIT0109] Qie S , Chu C, Li W et al ErbB2 activation upregulates glutaminase 1 expression which promotes breast cancer cell proliferation. J Cell Biochem 2014;115:498–509. 24122876 10.1002/jcb.24684 PMC4518873

[CIT0110] Qiu F , Chen YR, Liu X et al Arginine starvation impairs mitochondrial respiratory function in ASS1-deficient breast cancer cells. Sci Signal 2014;7:ra31. 24692592 10.1126/scisignal.2004761 PMC4229039

[CIT0111] Quek LE , van Geldermalsen M, Guan YF et al Glutamine addiction promotes glucose oxidation in triple-negative breast cancer. Oncogene 2022;41:4066–4078. 35851845 10.1038/s41388-022-02408-5 PMC9391225

[CIT0112] Rabinovich S , Adler L, Yizhak K et al Diversion of aspartate in ASS1-deficient tumours fosters de novo pyrimidine synthesis. Nature 2015;527:379–383. 26560030 10.1038/nature15529 PMC4655447

[CIT0113] Ramachandran S , S RS, Sharma M et al Expression and function of SLC38A5, an amino acid-coupled Na^+^/H^+^ exchanger, in triple-negative breast cancer and its relevance to macropinocytosis. Biochem J 2021;478:3957–3976. 34704597 10.1042/BCJ20210585 PMC8652584

[CIT0114] Reis LMD , Adamoski D, Ornitz Oliveira Souza R et al Dual inhibition of glutaminase and carnitine palmitoyltransferase decreases growth and migration of glutaminase inhibition-resistant triple-negative breast cancer cells. J Biol Chem 2019;294:9342–9357. 31040181 10.1074/jbc.RA119.008180 PMC6579458

[CIT0115] Reynolds MR , Lane AN, Robertson B et al Control of glutamine metabolism by the tumor suppressor Rb. Oncogene 2014;33:556–566. 23353822 10.1038/onc.2012.635 PMC3918885

[CIT0116] Robinson MM , McBryant SJ, Tsukamoto T et al Novel mechanism of inhibition of rat kidney-type glutaminase by bis-2-(5-phenylacetamido-1,2,4-thiadiazol-2-yl)ethyl sulfide (BPTES). Biochem J 2007;406:407–414. 17581113 10.1042/BJ20070039 PMC2049044

[CIT0117] Roci I , Watrous JD, Lagerborg KA et al Mapping metabolic events in the cancer cell cycle reveals arginine catabolism in the committed SG_2_M Phase. Cell Rep 2019;26:1691–1700.e5. 30759381 10.1016/j.celrep.2019.01.059 PMC6663478

[CIT0118] Rossi M , Altea-Manzano P, Demicco M et al PHGDH heterogeneity potentiates cancer cell dissemination and metastasis. Nature 2022;605:747–753. 35585241 10.1038/s41586-022-04758-2 PMC9888363

[CIT0119] Ruan X , Yan W, Cao M et al Breast cancer cell-secreted miR-199b-5p hijacks neurometabolic coupling to promote brain metastasis. Nat Commun 2024;15:4549. 38811525 10.1038/s41467-024-48740-0 PMC11137082

[CIT0120] Ruiu R , Rolih V, Bolli E et al Fighting breast cancer stem cells through the immune-targeting of the xCT cystine-glutamate antiporter. Cancer Immunol Immunother 2019;68:131–141. 29947961 10.1007/s00262-018-2185-1 PMC11028170

[CIT0121] Saito Y , Li L, Coyaud E et al LLGL2 rescues nutrient stress by promoting leucine uptake in ER_+_ breast cancer. Nature 2019;569:275–279. 30996345 10.1038/s41586-019-1126-2

[CIT0122] Sarangi P. Role of indoleamine 2, 3-dioxygenase 1 in immunosuppression of breast cancer. Cancer Pathog Ther 2024;2:246–255. 39371092 10.1016/j.cpt.2023.11.001 PMC11447360

[CIT0123] Scales TQ , Smith B, Blanchard LM et al A whole food, plant-based diet reduces amino acid levels in patients with metastatic breast cancer. Cancer Metab 2024;12:38. 39702320 10.1186/s40170-024-00368-w PMC11657127

[CIT0124] Schulte ML , Fu A, Zhao P et al Pharmacological blockade of ASCT2-dependent glutamine transport leads to antitumor efficacy in preclinical models. Nat Med 2018;24:194–202. 29334372 10.1038/nm.4464 PMC5803339

[CIT0125] Sennoune SR , Nandagopal GD, Ramachandran S et al Potent inhibition of Macropinocytosis by Niclosamide in cancer cells: a novel mechanism for the anticancer efficacy for the antihelminthic. Cancers (Basel) 2023;15:759. 36765717 10.3390/cancers15030759 PMC9913174

[CIT0126] Shahnazari M , Bigdeli R, Dashbolaghi A et al Biochemical and biological evaluation of an L-Asparaginase from isolated Escherichia coli MF-107 as an anti-tumor enzyme on MCF7 cell line. Iran Biomed J 2022;26:279–290. 35690915 10.52547/ibj.3494 PMC9432472

[CIT0127] Shajahan-Haq AN , Cook KL, Schwartz-Roberts JL et al MYC regulates the unfolded protein response and glucose and glutamine uptake in endocrine resistant breast cancer. Mol Cancer 2014;13:239. 25339305 10.1186/1476-4598-13-239 PMC4216870

[CIT0128] Sharma S , Singh B, Mishra AK et al LAT-1 based primary breast cancer detection by [99m]Tc-labeled DTPA-bis-methionine scintimammography: first results using indigenously developed single vial kit preparation. Cancer Biother Radiopharm 2014;29:283–288. 25203145 10.1089/cbr.2014.1655 PMC4158983

[CIT0129] Shen J , Yan L, Liu S et al Plasma metabolomic profiles in breast cancer patients and healthy controls: by race and tumor receptor subtypes. Transl Oncol 2013;6:757–765. 24466379 10.1593/tlo.13619 PMC3890711

[CIT0130] Shen X , Wang G, He H et al SLC38A5 promotes glutamine metabolism and inhibits cisplatin chemosensitivity in breast cancer. Breast Cancer 2024;31:96–104. 37914960 10.1007/s12282-023-01516-8

[CIT0131] Shi J , Pabon K, Ding R et al ABCG2 and SLC1A5 functionally interact to rewire metabolism and confer a survival advantage to cancer cells under oxidative stress. J Biol Chem 2024;300:107299. 38641063 10.1016/j.jbc.2024.107299 PMC11131071

[CIT0132] Soliman H , Khambati F, Han HS et al A phase-1/2 study of adenovirus-p53 transduced dendritic cell vaccine in combination with indoximod in metastatic solid tumors and invasive breast cancer. Oncotarget 2018;9:10110–10117. 29515795 10.18632/oncotarget.24118 PMC5839376

[CIT0133] Strekalova E , Malin D, Good DM et al Methionine deprivation induces a targetable vulnerability in triple-negative breast cancer cells by enhancing TRAIL Receptor-2 expression. Clin Cancer Res 2015;21:2780–2791. 25724522 10.1158/1078-0432.CCR-14-2792 PMC4470820

[CIT0134] Sullivan LB , Luengo A, Danai LV et al Aspartate is an endogenous metabolic limitation for tumour growth. Nat Cell Biol 2018;20:782–788. 29941931 10.1038/s41556-018-0125-0 PMC6051729

[CIT0135] Sullivan MR , Mattaini KR, Dennstedt EA et al Increased serine synthesis provides an advantage for tumors arising in tissues where serine levels are limiting. Cell Metab 2019;29:1410–1421.e4. 30905671 10.1016/j.cmet.2019.02.015 PMC6551255

[CIT0136] Sun N , Zhao X. Argininosuccinate synthase 1, arginine deprivation therapy and cancer management. Front Pharmacol 2022;13:935553. 35910381 10.3389/fphar.2022.935553 PMC9335876

[CIT0137] Sun X , Wang M, Wang M et al Metabolic reprogramming in triple-negative breast cancer. Front Oncol 2020;10:428. 32296646 10.3389/fonc.2020.00428 PMC7136496

[CIT0138] Switzer CH , Glynn SA, Cheng RY et al S-Nitrosylation of EGFR and Src activates an oncogenic signaling network in human basal-like breast cancer. Mol Cancer Res 2012;10:1203–1215. 22878588 10.1158/1541-7786.MCR-12-0124 PMC3463231

[CIT0139] Tade FI , Cohen MA, Styblo TM et al Anti-3-^18^F-fACBC (^18^F-Fluciclovine) PET/CT of breast cancer: an exploratory study. J Nucl Med 2016;57:1357–1363. 27056619 10.2967/jnumed.115.171389

[CIT0141] Tajan M , Vousden KH. Dietary approaches to cancer therapy. Cancer Cell 2020;37:767–785. 32413275 10.1016/j.ccell.2020.04.005

[CIT0140] Tajan M , Hennequart M, Cheung EC et al Serine synthesis pathway inhibition cooperates with dietary serine and glycine limitation for cancer therapy. Nat Commun 2021;12:366. 33446657 10.1038/s41467-020-20223-y PMC7809039

[CIT0142] Takaku H , Takase M, Abe S et al *In vivo* anti-tumor activity of arginine deiminase purified from Mycoplasma arginini. Int J Cancer 1992;51:244–249. 1568792 10.1002/ijc.2910510213

[CIT0143] Timmerman LA , Holton T, Yuneva M et al Glutamine sensitivity analysis identifies the xCT antiporter as a common triple-negative breast tumor therapeutic target. Cancer Cell 2013;24:450–465. 24094812 10.1016/j.ccr.2013.08.020 PMC3931310

[CIT0144] Tombari C , Zannini A, Bertolio R et al Mutant p53 sustains serine-glycine synthesis and essential amino acids intake promoting breast cancer growth. Nat Commun 2023;14:6777. 37880212 10.1038/s41467-023-42458-1 PMC10600207

[CIT0145] Tornroos R , Tina E, Gothlin Eremo A. SLC7A5 is linked to increased expression of genes related to proliferation and hypoxia in estrogen‑receptor‑positive breast cancer. Oncol Rep 2022;47:17. 34792178 10.3892/or.2021.8228 PMC8611404

[CIT0146] Triplett TA , Garrison KC, Marshall N et al Reversal of indoleamine 2,3-dioxygenase-mediated cancer immune suppression by systemic kynurenine depletion with a therapeutic enzyme. Nat Biotechnol 2018;36:758–764. 30010674 10.1038/nbt.4180 PMC6078800

[CIT0147] Tsuchiya T , Honda H, Oikawa M et al Oral administration of the amino acids cystine and theanine attenuates the adverse events of S-1 adjuvant chemotherapy in gastrointestinal cancer patients. Int J Clin Oncol 2016;21:1085–1090. 27306219 10.1007/s10147-016-0996-7 PMC5124434

[CIT0149] van Geldermalsen M , Wang Q, Nagarajah R et al ASCT2/SLC1A5 controls glutamine uptake and tumour growth in triple-negative basal-like breast cancer. Oncogene 2016;35:3201–3208. 26455325 10.1038/onc.2015.381 PMC4914826

[CIT0148] van Geldermalsen M , Quek LE, Turner N et al Benzylserine inhibits breast cancer cell growth by disrupting intracellular amino acid homeostasis and triggering amino acid response pathways. BMC Cancer 2018;18:689. 29940911 10.1186/s12885-018-4599-8 PMC6019833

[CIT0150] Vidal CM , Ouyang C, Qi Y et al Arginine regulates HSPA5/BiP translation through ribosome pausing in triple-negative breast cancer cells. Br J Cancer 2023;129:444–454. 37386138 10.1038/s41416-023-02322-x PMC10403569

[CIT0151] Vidula N , Yau C, Rugo HS. Glutaminase (*GLS1*) gene expression in primary breast cancer. Breast Cancer 2023;30:1079–1084. 37679553 10.1007/s12282-023-01502-0

[CIT0152] Wang Q , Liberti MV, Liu P et al Rational design of selective allosteric inhibitors of PHGDH and serine synthesis with anti-tumor activity. Cell Chem Biol 2017;24:55–65. 28042046 10.1016/j.chembiol.2016.11.013 PMC5915676

[CIT0153] Wang R , Xiang W, Xu Y et al Enhanced glutamine utilization mediated by SLC1A5 and GPT2 is an essential metabolic feature of colorectal signet ring cell carcinoma with therapeutic potential. Ann Transl Med2020a;8:302. 32355746 10.21037/atm.2020.03.31 PMC7186745

[CIT0154] Wang Z , Liu F, Fan N et al Targeting glutaminolysis: new perspectives to understand cancer development and novel strategies for potential target therapies. Front Oncol2020b;10:589508. 33194749 10.3389/fonc.2020.589508 PMC7649373

[CIT0155] Wei Z , Liu X, Cheng C et al Metabolism of amino acids in cancer. Front Cell Dev Biol 2020;8:603837. 33511116 10.3389/fcell.2020.603837 PMC7835483

[CIT0156] Wichmann CW , Burvenich IJG, Guo N et al Preclinical radiolabeling, in vivo biodistribution and positron emission tomography of a novel pyrrolobenzodiazepine (PBD)-based antibody drug conjugate targeting ASCT2. Nucl Med Biol 2023;122–123:108366. 10.1016/j.nucmedbio.2023.108366 37473513

[CIT0157] Wicker CA , Hunt BG, Krishnan S et al Glutaminase inhibition with telaglenastat (CB-839) improves treatment response in combination with ionizing radiation in head and neck squamous cell carcinoma models. Cancer Lett 2021;502:180–188. 33450358 10.1016/j.canlet.2020.12.038 PMC7897292

[CIT0158] Wu Q , Li J, Sun S et al YAP/TAZ-mediated activation of serine metabolism and methylation regulation is critical for LKB1-deficient breast cancer progression. Biosci Rep 2017;37:BSR20171072. 28931725 10.1042/BSR20171072 PMC5653917

[CIT0159] Xie G , Zhou B, Zhao A et al Lowered circulating aspartate is a metabolic feature of human breast cancer. Oncotarget 2015;6:33369–33381. 26452258 10.18632/oncotarget.5409 PMC4741772

[CIT0160] Xu X , Zhu H, Liu F et al Dynamic PET/CT imaging of ^18^F-(2S, 4R)4-fluoroglutamine in healthy volunteers and oncological patients. Eur J Nucl Med Mol Imaging 2020;47:2280–2292. 32166510 10.1007/s00259-019-04543-w

[CIT0161] Xue C , Li G, Zheng Q et al Tryptophan metabolism in health and disease. Cell Metab 2023;35:1304–1326. 37352864 10.1016/j.cmet.2023.06.004

[CIT0164] Yang TS , Lu SN, Chao Y et al A randomised phase II study of pegylated arginine deiminase (ADI-PEG 20) in Asian advanced hepatocellular carcinoma patients. Br J Cancer 2010;103:954–960. 20808309 10.1038/sj.bjc.6605856 PMC2965867

[CIT0165] Yang WS , SriRamaratnam R, Welsch ME et al Regulation of ferroptotic cancer cell death by GPX4. Cell 2014;156:317–331. 24439385 10.1016/j.cell.2013.12.010 PMC4076414

[CIT0162] Yang J , Zhou Y, Xie S et al Metformin induces Ferroptosis by inhibiting UFMylation of SLC7A11 in breast cancer. J Exp Clin Cancer Res2021a;40:206. 34162423 10.1186/s13046-021-02012-7 PMC8223374

[CIT0163] Yang R , Guo Z, Zhao Y et al Compound 968 reverses adriamycin resistance in breast cancer MCF-7(ADR) cells via inhibiting P-glycoprotein function independently of glutaminase. Cell Death Discov2021b;7:204. 34354052 10.1038/s41420-021-00590-1 PMC8342604

[CIT0166] Yao S , Janku F, Koenig K et al Phase 1 trial of ADI-PEG 20 and liposomal doxorubicin in patients with metastatic solid tumors. Cancer Med 2022;11:340–347. 34841717 10.1002/cam4.4446 PMC8729058

[CIT0167] Yoo HC , Yu YC, Sung Y et al Glutamine reliance in cell metabolism. Exp Mol Med 2020;52:1496–1516. 32943735 10.1038/s12276-020-00504-8 PMC8080614

[CIT0168] You M , Xie Z, Zhang N et al Signaling pathways in cancer metabolism: mechanisms and therapeutic targets. Signal Transduct Target Ther 2023;8:196. 37164974 10.1038/s41392-023-01442-3 PMC10172373

[CIT0169] Zhang X , Bai W. Repression of phosphoglycerate dehydrogenase sensitizes triple-negative breast cancer to doxorubicin. Cancer Chemother Pharmacol 2016;78:655–659. 27473325 10.1007/s00280-016-3117-4

[CIT0170] Zhang Y , Lindstrom S, Kraft P et al Genetic risk, health-associated lifestyle, and risk of early-onset total cancer and breast cancer. J Natl Cancer Inst 2025;117:40–48. 39189966 10.1093/jnci/djae208 PMC11717420

[CIT0171] Zhou LY , Shi YH, Jia YS et al Potential role of pemetrexed in metastatic breast cancer patients pre-treated with anthracycline or taxane. Chronic Dis Transl Med 2015;1:27–35. 29062984 10.1016/j.cdtm.2015.02.008 PMC5643787

[CIT0173] Zhou X , Zheng W, Nagana Gowda GA et al 1,25-Dihydroxyvitamin D inhibits glutamine metabolism in Harvey-ras transformed MCF10A human breast epithelial cell. J Steroid Biochem Mol Biol 2016;163:147–156. 27154413 10.1016/j.jsbmb.2016.04.022 PMC5012911

[CIT0172] Zhou R , Pantel AR, Li S et al [^18^F](2S,4R)4-Fluoroglutamine PET detects glutamine pool size changes in triple-negative breast cancer in response to glutaminase inhibition. Cancer Res 2017;77:1476–1484. 28202527 10.1158/0008-5472.CAN-16-1945 PMC5362115

